# Adulthood stressful life events as predictors of incident cardiovascular disease: insights from two prospective cohorts

**DOI:** 10.1186/s12916-026-04890-0

**Published:** 2026-04-24

**Authors:** Shuang Wu, Siqi Lyu, Yimeng Wang, Hanyang Liang, Wei Xu, Juan Wang, Xinghui Shao, Han Zhang, Hongyu Liu, Bi Huang, Yang Chen, Gregory Y. H. Lip

**Affiliations:** 1 National Center for Cardiovascular Disease, Fuwai Hospital, Chinese Academy of Medical Sciences and Peking Union Medical College, Beijing, People’s Republic of China; 2 National Clinical Research Center of Cardiovascular Diseases, Fuwai Hospital, National Center for Cardiovascular Disease, Chinese Academy of Medical Sciences, Peking Union Medical College, Beijing, People’s Republic of China; 3 Liverpool Centre for Cardiovascular Science, University of Liverpool, Liverpool John Moores University and Liverpool Heart and Chest Hospital, William Henry Duncan Building, 6 West Derby Street, Liverpool, L7 8TX UK; 4 Department of Cardiovascular Medicine, the Second Affiliated Hospital, Jiangxi Medical College, Nanchang University, Nanchang, Jiangxi People’s Republic of China; 5 Department of Cardiology, The First Affiliated Hospital of Chongqing Medical University, Chongqing, People’s Republic of China; 6 Department of Cardiovascular and Metabolic Medicine, Institute of Life Course and Medical Sciences, University of Liverpool, Liverpool, UK; 7 Danish Centre for Health Services Research, Department of Clinical Medicine, Aalborg University, Aalborg, Denmark; 8 Medical University of Bialystok, Bialystok, Poland

**Keywords:** stressful life events, cardiovascular disease, heart disease, stroke, mediation analysis, population-attributable fraction

## Abstract

**Background:**

Stressful life events (SLEs) in adulthood are potential risk factors for cardiovascular disease (CVD), yet evidence remains inconsistent. This study examined the association between adulthood SLEs and incident CVD and evaluated mediation by depression, physical inactivity, and smoking.

**Methods:**

We analyzed harmonized data from two nationally representative aging cohorts: the US Health and Retirement Study and the English Longitudinal Study of Ageing. SLEs were assessed at baseline, and incident CVD was physician-diagnosed. We used Cox and restricted mean survival time (RMST) regression to estimate hazard ratios (HRs) and RMST differences, set at the time point when 90% of incident CVD events had accrued (7.8 years). Population attributable fractions (PAFs) based on RMST and mediation analyses via RMST pseudo-value regression quantified contributions of SLEs and behavioral pathways.

**Results:**

Among 18,898 participants without baseline cardiovascular disease (mean age 64.5 years; 39.8% male), 2,782 incident CVD cases were documented (incidence rate: 2.51 per 100 person-years). RMST analysis showed that any SLE exposure was related to significant reductions in event-free survival, including a 2.22-month decrease for CVD (95% CI: -2.90 to -1.53), a 1.57-month decrease for heart disease (95% CI: -2.16 to -0.99), and a 0.79-month decrease for stroke (95% CI: -1.16 to -0.41), with a clear dose-response relationship. Corresponding Cox proportional hazards models showed adjusted HRs of 1.20 (95% CI: 1.11–1.30) for CVD, 1.16 (95% CI: 1.07–1.27) for heart disease, and 1.26 (95% CI: 1.09–1.46) for stroke. Each additional SLE increased CVD, heart disease, and stroke risks by 11%, 10%, and 11%. PAFs attributable to SLEs exposure were 2.6% for CVD, 1.7% for heart disease, and 0.8% for stroke. Mediation analyses revealed that depression, physical inactivity, and current smoking accounted for 7.2%, 4.5%, and 4.5% of the total effect of SLEs on CVD, respectively.

**Conclusions:**

Adulthood SLEs are independently associated with increased CVD risk, with depression, physical inactivity, and smoking explaining a modest proportion of this association.

**Supplementary Information:**

The online version contains supplementary material available at 10.1186/s12916-026-04890-0.

## Background

Globally, cardiovascular diseases (CVDs), particularly ischemic heart disease and stroke, are the leading causes of mortality and significantly contribute to the burden of disability [1, 2]. The burden of CVD disability manifests through two primary pathways: acute events that lead to permanent functional impairment (e.g., 50% of stroke survivors experience long-term disability [3]), and chronic progression that results in escalating activity limitations. This trend carries significant socioeconomic implications, with the economic burden of CVD in the European Union estimated at €282 billion annually. Health and long-term care costs account for €155 billion of this total, representing 11% of overall European Union health expenditure [4]. In the United States, annual healthcare costs are projected to rise from $393 billion in 2020 to $1,490 billion by 2050 [5].

Emerging evidence suggests that psychosocial stressors serve as modifiable risk amplifiers for CVD risk [6, 7]. Adverse childhood events, such as physical abuse, interparental physical violence, household substance abuse, etc., have been identified as risk factors for multiple chronic conditions, including CVD during adulthood [6, 8]. Compared to childhood stress and traditional adult risk factors like smoking, high blood pressure, and high blood glucose levels, the detrimental effects of stress during adulthood are generally less marked. Stressful life events (SLEs) are defined as discrete, observable occurrences that substantially disrupt an individual’s psychosocial stability, necessitating significant cognitive and behavioral adaptation. These events can induce considerable emotional distress, often exceeding an individual’s immediate coping resources. Operationally, SLEs encompass a range of adverse experiences, including but not limited to: (1) interpersonal losses (e.g., bereavement, marital dissolution), (2) socioeconomic adversities (e.g., unemployment, asset deprivation), (3) health-related crises (e.g., life-threatening diagnoses, traumatic injuries), and (4) role transitions (e.g., caregiver burden, family estrangement) [9]. Existing studies on the association between exposure to SLEs during adulthood and the risk of CVD, including heart disease and stroke, have yielded inconsistent conclusions [10].

A growing body of evidence suggests that psychosocial stress may contribute to CVD risk not only directly but also indirectly through modifiable behavioral and psychological pathways. Specifically, exposure to significant stress is linked to the development and exacerbation of depression, which, in turn, is associated with physiological dysregulations such as enhanced systemic inflammation, endothelial dysfunction, and autonomic imbalance [11]. These collectively promote atherogenesis and increase vulnerability to cardiovascular events. Besides, chronic stress often precipitates maladaptive coping responses, notably reduced physical activity and increased smoking, both of which are well-established independent risk factors for CVD [12, 13]. Taken together, this evidence indicates that depressive symptoms, physical inactivity, and smoking may constitute important mediating pathways linking SLEs to CVD onset. However, the extent to which these factors mediate the association between SLEs and incident CVD in older adults remains to be established.

To address this issue, we employ harmonized data from two nationally representative aging cohorts, the primary hypothesis of this research posits that exposure to SLEs during adulthood is significantly associated with an increased risk of developing CVD, independent of traditional risk factors such as smoking, hypertension, and diabetes. Second, we explore the mediating roles of depressive symptoms, physical inactivity, and smoking in the association between adulthood SLEs and CVD risk.

## Methods

### Study design and population

For this analysis, we utilized individual-level data from two nationally representative longitudinal studies: the US Health and Retirement Study (HRS) and the English Longitudinal Study on Ageing (ELSA). Both studies employ harmonized methodologies that facilitate cross-national comparisons of aging processes, with detailed study designs published elsewhere [14, 15]. Briefly, the HRS was initiated in 1992 and follows a biennial survey design to track health, economic, and social transitions among American adults aged 50 and older. Similarly, the ELSA was established in 2002 and employs a parallel biennial design to collect comparable longitudinal data on physical and mental health, wealth dynamics, and later-life outcomes in the UK. The HRS received approval from the University of Michigan, while the ELSA obtained approval from the London Multi-Centre Research Ethics Committee. Informed consent was obtained from all participants in these cohorts. The study followed the Strengthening the Reporting of Observational Studies in Epidemiology reporting guidelines.

We used data from waves 11–15 (2012–2020) for the HRS and waves 3–8 (2006–2016) for the ELSA. Baseline was defined as wave 11 for HRS and wave 3 for ELSA. In primary analyses, mediators were assessed at baseline (wave 11 for HRS and wave 3 for ELSA). In sensitivity analyses, mediators were assessed at the first follow-up wave (wave 12 for HRS and wave 4 for ELSA) to mitigate potential reverse causation. Participants were eligible for inclusion if they met the following criteria: (1) documented information on adulthood SLEs; (2) no known history of CVD at baseline; and (3) provided information on CVD outcomes during follow-up. Based on these criteria, a total of 18,898 participants were included in the primary analysis, comprising 13,366 from the HRS and 5,532 from the ELSA (Additional file: Fig. S1).

### Assessment of adulthood SLEs

Adulthood SLEs was assessed using established methodologies from prior research and incorporated six specific adverse events: (1) unemployment, (2) asset poverty, (3) death of a child, (4) death of a spouse or partner, (5) life-threatening illness or accident, and (6) physical attack or injury [16]. Data were collected through structured interviews and comprehensive life course assessments (Additional file: Table S1). Specifically, unemployment was defined as being involuntarily unemployed and actively seeking work at the baseline assessment; retired respondents were not considered unemployed. Asset poverty was defined as having zero or negative net worth at baseline. Both measures captured point-in-time exposures.

Each event was dichotomously coded (present = 1, absent = 0). A cumulative SLE score was then calculated by summing these binary indicators. For analysis, exposure was operationalized in three complementary forms: (1) as a continuous cumulative score, (2) as a binary variable (no SLEs vs. any SLEs [≥ 1]), and (3) as a three-category variable (none, moderate [1, 2], and high [> 2] SLEs). The three-category variable was specifically created to examine the dose-response relationship across increasing levels of exposure.

### Follow-up and definition of outcomes

Follow-up continued until the first occurrence of any endpoint event: the first diagnosis of CVD, death, or the end of follow-up, whichever occurred first. The primary outcome was incident CVD, defined as a composite endpoint that included physician-diagnosed myocardial infarction, angina pectoris, congestive heart failure, other heart diseases, and stroke. Secondary outcomes included heart diseases (including myocardial infarction, angina pectoris, congestive heart failure, and other heart conditions) and stroke. Case ascertainment was based on participants’ self-reported physician diagnoses obtained through standardized questionnaires. Participants were classified as CVD cases if they reported any of the specified cardiovascular conditions during follow-up assessments.

### Assessments of covariates

Demographic and health-related data were collected through face-to-face interviews. The following covariates were collected from both cohorts: age, sex (male or female), marital status (married/partnered or other marital statuses), educational attainment (below high school, high school, college or above), current smoking, current alcohol consumption, physical activity level (light level, moderate level, and vigorous level), body mass index (BMI), depressive symptoms, self-reported physician-diagnosed comorbidities (hypertension and diabetes), and medications (including anti-hypertensive and anti-diabetic drugs). Current alcohol consumption was defined as ≥ 1 drink per drinking day in the past three months in HRS, and as drinking at any frequency over the past 12 months in ELSA. BMI was calculated as weight in kilograms divided by height in meters squared. Depressive symptoms were measured via the 8-item Center for Epidemiologic Studies Depression Scale (CES-D-8). A total score of 3 or higher was used to indicate depression [17]. A comprehensive overview of variable definitions, source items, original coding, and harmonization rules across HRS and ELSA is provided in Additional file: Table S2.

### Statistical analysis

All statistical analyses were performed using R software (version 4.3.1; R Foundation for Statistical Computing, Vienna, Austria). A two-tailed p-value < 0.05 was considered statistically significant. CES-D-8 score, BMI, and educational attainment exhibited the highest proportions of missing data (Additional file: Table S3). We handled missing covariate data using multiple imputation by chained equations in the main analysis. Baseline characteristics are presented according to SLEs exposure categories. Continuous variables are reported as mean ± standard deviation and compared using analysis of variance. Categorical variables are reported as numbers (percentages) and compared using the chi‑square tests.

Cox proportional hazards regression models were initially employed to estimate hazard ratios (HRs) and 95% confidence intervals (CIs) for the associations between adulthood SLEs and incident cardiovascular outcomes. Two models were constructed: Model 1 was unadjusted, and Model 2 was adjusted for age, sex, education, current drinking, BMI, hypertension, and diabetes. Since the proportional hazards assumption was violated based on Schoenfeld residuals, we complemented the Cox analysis with Restricted Mean Survival Time (RMST) analysis, which does not rely on this assumption and provides an intuitive measure of survival difference. RMST represents the average event-free survival time within a specified time window. The truncation time for RMST was set at τ = 7.8 years, corresponding to the time when 90% of incident CVD events had occurred. This time point provides stable estimation by utilizing the period of maximal event accrual before risk-set diminution. RMST differences (ΔRMST) and 95% CIs were calculated using the survRM2 package, which implements the Kaplan-Meier-based method [18]. Both Cox and RMST results are presented together to offer complementary insights into the association between SLE exposure and cardiovascular risk.

In the main analyses, hypertension, diabetes, BMI, and alcohol consumption were included as covariates rather than mediators. Although these factors may theoretically lie on the causal pathway between SLEs and CVD, they are situated downstream of depression, smoking, and physical inactivity in the causal pathway. Because hypertension, diabetes, and BMI are strongly associated with CVD outcomes, treating them as mediators would likely absorb much of the effect of upstream behavioral factors, potentially underestimating the behavioral and psychological pathways of interest. We therefore included them as confounders to avoid over-adjustment. Alcohol consumption was similarly treated as a confounder given its well-documented J-shaped association with CVD, which complicates its interpretation as a simple mediator.

We conducted causal mediation analyses using RMST pseudo-value regression to examine the mediating roles of depression, physical inactivity, and smoking in the relationship between adulthood SLEs and cardiovascular outcomes. For each cardiovascular outcome, individual pseudo-values were computed at the same truncation time (τ = 7.8 years) using the Andersen and Perme method [19]. We then specified a three‑equation structural model to decompose the total effect of SLE exposure into its direct and indirect (mediated) components: (1) a total effect model regressed the RMST pseudo‑value on SLE exposure; (2) a mediator model estimated the association between SLE exposure and each mediator; (3) an outcome model regressed the RMST pseudo‑value on both SLE exposure and the mediator. All variables were operationalized as binary variables in these models (any SLE vs. none; depression vs. no depression; less than vigorous vs. vigorous physical activity; current smoker vs. non-smoker), and analyses were adjusted for the same covariates as in Model 2. The indirect (mediated) effect was quantified as the product of the coefficient for SLE exposure in the mediator model and the coefficient for the mediator in the outcome model. We calculated the Percentage of Excess Risk Mediated (PERM) for each pathway as (indirect effect / total effect) × 100%.

In parallel, we quantified the population burden of SLEs and each mediator using time-dependent population-attributable fractions (PAFs) based on RMST differences, maintaining the same covariate adjustments as in Model 2. For each exposure (binary SLEs and each mediator) and each outcome (CVD, heart disease, stroke), we performed non-parametric RMST estimation via the rmst2() function from the “survRM2” package. PAF at time τ was computed as:

$$\begin{array}{lll}{\mathrm{PAF}}_{{(\tau)}} && = \left(\frac{{\mathrm{RMST}}_{{\mathrm{unexposed}}(\tau)}{-}{\mathrm{RMST}}_{{\mathrm{exposed}}(\tau)}}{{\mathrm{RMST}}_{\mathrm{unexposed}}(\tau)}\right) \\ && \times 100\% \end{array}$$

Analyses were conducted at 18 follow-up time points at 6-month intervals (0, 6, 12, …, 108 months) to visualize how attributable risk changed over the study period.

We conducted a series of sensitivity analyses to evaluate the robustness of our primary findings. These included: repeating main analyses with cohort-stratified analyses for HRS and ELSA; performing a mediation analysis using second-wave mediator data to mitigate potential reverse causation; repeating main analyses using complete‑case data not being imputed; further adjusting for anti‑hypertensive and anti‑diabetic medications; accounting for competing risk of death via Fine‑Gray subdistribution hazard models; excluding participants with incident CVD in the first two years of follow‑up to mitigate reverse causation; and restricting the sample to reduce self‑report misclassification by excluding participants aged ≥ 75 years, with education below high school, or cognitive score in the lowest quartile. To explore heterogeneity across SLE types, we performed event-specific Cox and RMST models and constructed a weighted SLE score based on each event’s association with baseline depressive symptoms (CES-D-8), with weights derived from standardized regression coefficients (see Supplemental Material: Construction of the Weighted Stressful Life Events Score and Reanalysis). Subgroup analyses were performed by age (< 65 vs. ≥65 years) and sex, with multiplicative interaction tested using product terms in Cox regression.

## Results

### Baseline characteristics of the study population

Among the 18,898 participants (mean age 64.5 ± 10.9 years; 39.8% male), we observed significant differences across SLEs exposure groups (Table 1). Participants reporting 1–2 SLEs were older than those with no SLEs or > 2 SLEs. We identified a socioeconomic gradient: individuals with > 2 SLEs had the highest proportion of below‑high‑school education and the lowest marriage/partnership rate. Health behaviors showed a graded association with SLEs burden: current smoking prevalence nearly doubled from the no‑SLEs group (12.9%) to the > 2‑SLEs group (27.2%), while vigorous physical activity decreased from 52.6% to 31.4%. Clinical profiles followed a similar pattern; compared to the no‑SLEs group, the > 2 SLEs group exhibited higher BMI, more severe depressive symptoms, and greater hypertension prevalence. These patterns remained consistent in cohort‑specific analyses of HRS and ELSA participants (Additional file: Table S4).

**Table 1:** Characteristics of pooled individual participant data from tow cohorts stratified by adulthood SLEs exposure

Variables	All (*n* = 18898)	No SLEs (*n* = 11215)	1–2 SLEs (*n* = 7396)	> 2 SLEs (*n* = 287)	*P* value
Age, mean (SD), years	64.52 ± 10.90	63.40 ± 9.89	66.23 ± 12.03	64.33 ± 12.15	< 0.001
Sex, n (%)					< 0.001
Male	7525(39.82)	4847(43.22)	2586(34.96)	92(32.06)	
Female	11,373(60.18)	6368(56.78)	4810(65.04)	195(67.94)	
Education, n (%)					< 0.001
Below high school	4144(21.93)	1906(17.00)	2135(28.87)	103(35.89)	
High school	7148(37.82)	4167(37.16)	2885(39.01)	96(33.45)	
College or above	7606(40.25)	5142(45.85)	2376(32.13)	88(30.66)	
Marital status, n (%)					< 0.001
**Married or partnered**	12,675(67.07)	9178(81.84)	3416(46.19)	81(28.22)	
**Other marital status**	6223(32.93)	2037(18.16)	3980(53.81)	206(71.78)	
Current smoking, n (%)	2772(14.67)	1442(12.86)	1252(16.93)	78(27.18)	< 0.001
Current drinking, n (%)	12,666(67.02)	7769(69.27)	4722(63.85)	175(60.98)	< 0.001
Physical activity, n (%)					< 0.001
Light level	2820(14.92)	1300(11.59)	1447(19.56)	73(25.44)	
Moderate level	7071(37.42)	4017(35.82)	2930(39.62)	124(43.21)	
Vigorous level	9007(47.66)	5898(52.59)	3019(40.82)	90(31.36)	
BMI, mean (SD), kg/m2	28.36 ± 5.83	28.21 ± 5.60	28.56 ± 6.11	29.51 ± 6.87	< 0.001
CESD, mean (SD)	1.37 ± 1.91	1.09 ± 1.68	1.75 ± 2.13	2.63 ± 2.46	< 0.001
Hypertension, n (%)	8877(46.97)	5098(45.46)	3634(49.13)	145(50.52)	< 0.001
Diabetes, n (%)	2994(15.84)	1701(15.17)	1241(16.78)	52(18.12)	< 0.010
Antihypertensive drugs, n (%)	7762(41.07)	4438(39.57)	3200(43.27)	124(43.21)	< 0.001
Antidiabetic drugs, n (%)	2427(12.84)	1375(12.26)	1007(13.62)	45(15.68)	< 0.010

BMI, body mass index; CESD, Center for Epidemiologic Studies Depression Scale; SD, Standard Deviation; SLEs, stressful life events.

### Exposure to adulthood SLEs and incident CVD

**Fig. 1:**
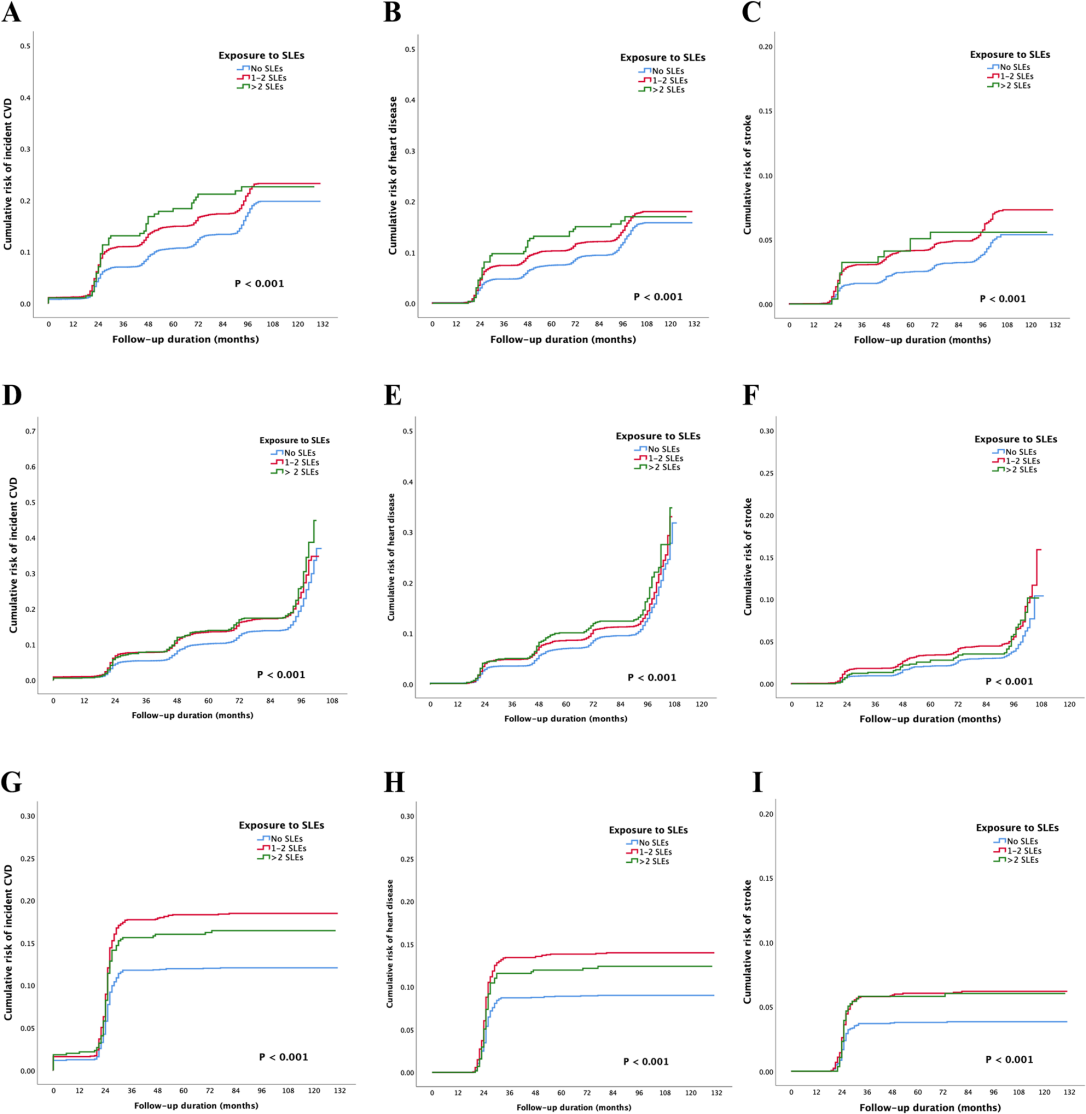
Kaplan-Meier survival curves for cardiovascular outcomes across different adulthood SLEs exposure categories. Overall participants: (**A**) Cumulative risk of incident CVD, (**B**) Cumulative risk of heart disease, (**C**) Cumulative risk of stroke; Participants from HRS: (**D**) Cumulative risk of incident CVD, (**E**) Cumulative risk of heart disease, (**F**) Cumulative risk of stroke; Participants from ELSA: (**G**) Cumulative risk of incident CVD, (**H**) Cumulative risk of heart disease, (**I**) Cumulative risk of stroke; Log-rank P values are shown for each panel CVD, Cardiovascular Disease; ELSA, English Longitudinal Study of Ageing; HRS, Health and Retirement Study; SLEs, stressful life events. The curves showed distinct patterns between cohorts: the stepwise increase in HRS reflects its biennial interview schedule, while the plateau in ELSA is likely attributable to greater censoring in later follow-up years. Despite these differences, SLE exposure was significantly associated with higher risk of all outcomes in both cohorts (log-rank *P* < 0.001 for all)

During a median follow-up of 90 months (interquartile range 33–96 months), 2,782 incident CVD cases (2.51 per 100 person-years) occurred, including 2,226 cases of heart disease (2.01 per 100 person-years) and 796 strokes (0.72 per 100 person-years). The cumulative incidence of all outcomes was higher among participants with SLEs compared with those without SLEs (Table 2). Kaplan-Meier curves depicted these differences, with log-rank tests confirming statistically significant separation among exposure groups (Fig. 1).

**Table 2:** Restricted mean survival time and Cox proportional hazards model analyses evaluating the impact of adulthood SLEs exposure on risks of cardiovascular outcomes

All participants	Cardiovascular disease					Heart disease					Stroke			
	Event rate (events/100 person years)	RMST difference (95% CI)	Cox models			Event rate (events/100 person years)	RMST difference (95% CI)	Cox models			Event rate (events/100 person years)	RMST difference (95% CI)	Cox models	
			HR (95% CI )	*P* value				HR (95% CI )	*P* value				HR (95% CI )	*P* value
**Model 1**														
Binary SLEs														
No SLEs	2.24	1(reference)	1(reference)			1.83	1(reference)	1(reference)			0.60	1(reference)	1(reference)	
SLEs	2.91	-2.97 (-3.65, -2.30)	1.30 (1.21, 1.40)	< 0.001		2.27	-2.05 (-2.62, -1.48)	1.24 (1.14, 1.35)	< 0.001		0.89	-1.17 (-1.54, -0.81)	1.48 (1.29, 1.70)	< 0.001
Categorical SLEs														
No SLEs	2.24	1(reference)	1(reference)			1.83	1(reference)	1(reference)			0.60	1(reference)	1(reference)	
1–2 SLEs	2.90	-2.90 (-3.58, -2.22)	1.30 (1.20, 1.40)	< 0.001		2.26	-1.98 (-2.56, -1.40)	1.23 (1.13, 1.34)	< 0.001		0.90	-1.17 (-1.54, -0.79)	1.48 (1.29, 1.70)	< 0.001
SLEs > 2	3.27	-4.79 (-7.51, -2.06)	1.45 (1.10, 1.93)	0.009		2.62	-3.82 (-6.14, -1.50)	1.37 (1.00, 1.88)	0.048		0.85	-1.42 (-2.91, 0.07)	1.37 (0.79, 2.39)	0.260
Continuous SLEs														
Per additional SLE	2.51	-1.82 (-2.29, -1.35)	1.17 (1.11, 1.22)	< 0.001		2.01	-1.32 (-1.72, -0.92)	1.15 (1.08, 1.21)	< 0.001		0.72	-0.67 (-0.93, -0.42)	1.23 (1.13, 1.34)	< 0.001
**Model 2**														
Binary SLEs														
No SLEs	2.24	1(reference)	1(reference)			1.83	1(reference)	1(reference)			0.60	1(reference)	1(reference)	
SLEs	2.91	-2.22 (-2.90, -1.53)	1.20 (1.11, 1.30)	< 0.001		2.27	-1.57 (-2.16, -0.99)	1.16 (1.07, 1.27)	< 0.001		0.89	-0.79 (-1.16, -0.41)	1.26 (1.09, 1.46)	0.002
Categorical SLEs														
No SLEs	2.24	1(reference)	1(reference)			1.83	1(reference)	1(reference)			0.60	1(reference)	1(reference)	
1–2 SLEs	2.90	-2.13 (-2.82, -1.44)	1.20 (1.11, 1.29)	< 0.001		2.26	-1.50 (-2.08, -0.90)	1.16 (1.06, 1.26)	< 0.001		0.90	-0.77 (-1.15, -0.39)	1.26 (1.09, 1.45)	0.002
SLEs > 2	3.27	-4.42 (-7.12, -1.71)	1.38 (1.04, 1.84)	0.025		2.62	-3.58 (-5.89, -1.27)	1.31 (0.96, 1.80)	0.092		0.85	-1.18 (-2.67, 0.31)	1.20 (0.69, 2.10)	0.513
Continuous SLEs														
Per additional SLE	2.51	-1.33 (-1.81, -0.86)	1.11 (1.05, 1.17)	< 0.001		2.01	-1.01 (-1.41, -0.61)	1.10 (1.04, 1.17)	0.001		0.72	-0.42 (-0.68, -0.16)	1.11 (1.01, 1.22)	0.029

Model 1 was unadjusted

Model 2 was adjusted for age, sex, education, current drinking status, body mass index, hypertension, and diabetes

CI, confidence interval; ELSA, English Longitudinal Study of Ageing; HR, hazard ratio; HRS, Health and Retirement Study; RMST, restricted mean survival time; SLEs, stressful life events

RMST analyses revealed substantial survival time deficits associated with SLEs exposure. In the fully adjusted model (Model 2), participants exposed to any SLEs experienced a 2.22-month shorter CVD free survival (95% CI: -2.90 to -1.53), a 1.57-month shorter heart disease free survival (95% CI: -2.16 to -0.99), and a 0.79-month shorter stroke free survival (95% CI: -1.16 to -0.41) over the 7.8-year observation period compared to those without exposure. A clear dose-response relationship was observed. Relative to the unexposed group, individuals with 1–2 SLEs showed RMST deficits of 2.13 months for CVD, 1.50 months for heart disease, and 0.77 months for stroke. Those with > 2 SLEs exhibited substantially larger deficits: 4.42 months for CVD, 3.58 months for heart disease, and 1.18 months for stroke. When modeled continuously, each additional SLE was associated with a 1.33-month reduction in CVD free survival (95% CI: -1.81 to -0.86), a 1.01-month reduction in heart disease free survival (95% CI: -1.41 to -0.61), and a 0.42-month reduction in stroke free survival (95% CI: -0.68 to -0.16). Corresponding Cox model results were consistent with the RMST findings. Any SLEs exposure was associated with increased hazards for CVD (aHR = 1.20, 95% CI: 1.11–1.30), heart disease (aHR = 1.16, 95% CI: 1.07–1.27), and stroke (aHR = 1.26, 95% CI: 1.09–1.46). Cohort-stratified analyses showed generally similar patterns (Additional file: Table S5).

### PAFs and mediation of sles and cardiovascular risk factors

**Fig. 2:**
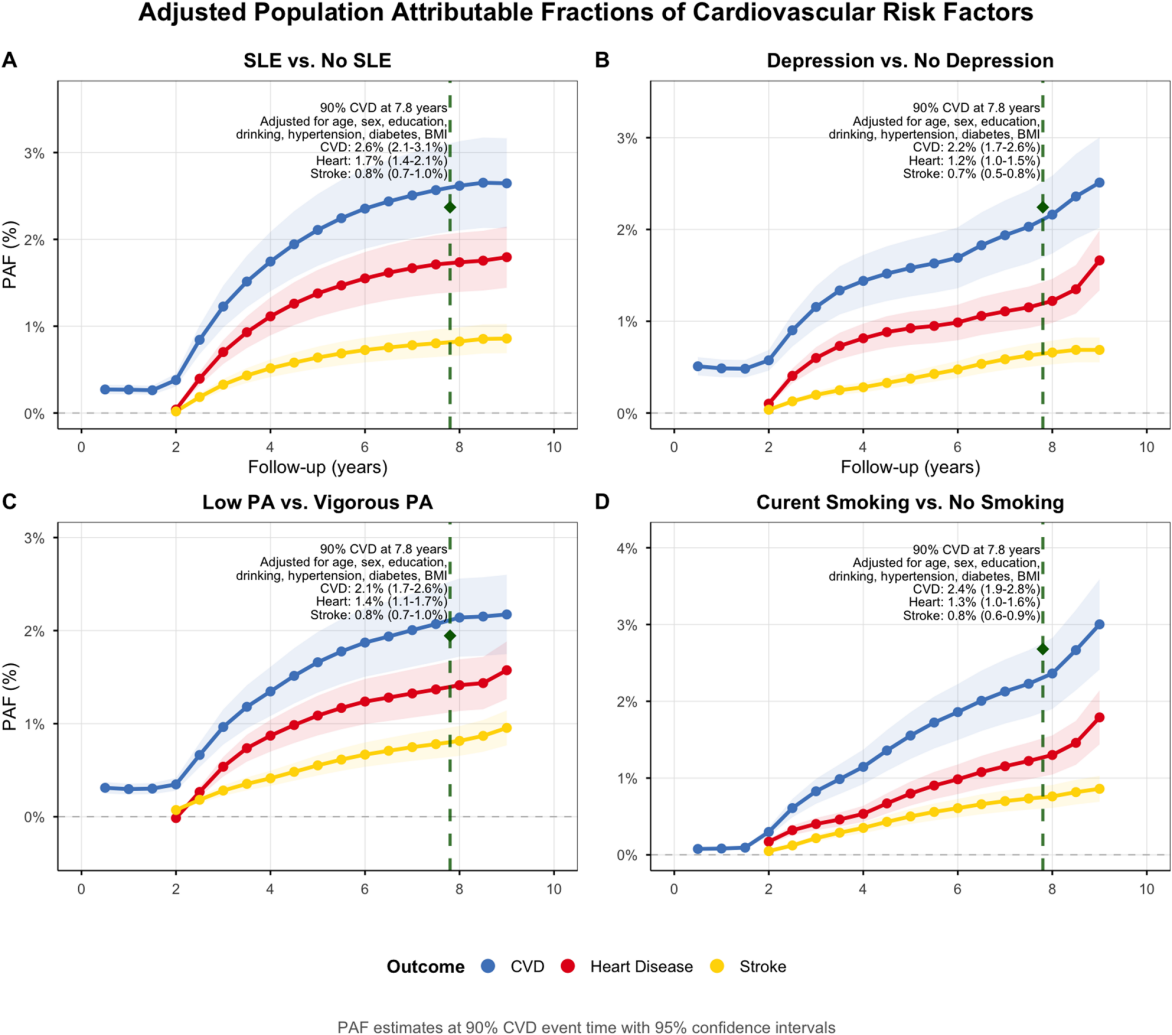
Population attributable fractions for incident CVD associated with adulthood SLEs and potential mediators (depression, physical inactivity, and current smoking).Models were adjusted for age, sex, education, drinking, hypertension, diabetes, and BMI. The PAF curves illustrate the proportion of incident CVD that could be attributed to each exposure over follow-up time. Shaded areas represent 95% confidence intervals. Vertical dashed line indicates the time point at which 90% of CVD events had occurred (7.8 years). Specific PAF estimates at 7.8 years are presented with 95% CIs for total CVD, heart disease, and stroke. **A** PAF for CVD associated with SLE exposure (vs. no SLE). **B** PAF for CVD associated with depression (vs. no depression). **C** PAF for CVD associated with low physical activity (vs. vigorous physical activity). **D** PAF for CVD associated with current smoking (vs. no smoking) Abbreviations: CI, confidence interval; CVD, cardiovascular disease; PAF, population attributable fraction; SLEs, stressful life events

Figure 2 presents time-dependent PAFs for cardiovascular outcomes associated with adulthood SLEs and three behavioral mediators. In fully adjusted models, the PAF for SLEs was 2.6% (95% CI: 1.7–3.1%) for CVD, 1.7% (1.4–2.1%) for heart disease, and 0.8% (0.7-1.0%) for stroke. For depressive symptoms, PAFs were 2.2% (1.7–2.6%) for CVD, 1.2% (1.0-1.5%) for heart disease, and 0.7% (0.5–0.8%) for stroke. For physical inactivity, PAFs were 2.1% (1.7–2.6%) for CVD, 1.4% (1.1–1.7%) for heart disease, and 0.8% (0.7-1.0%) for stroke. For current smoking, PAFs were 2.4% (1.9–2.8%) for CVD, 1.3% (1.0-1.6%) for heart disease, and 0.8% (0.6–0.9%) for stroke.

Table 3 summarizes the mediation analyses based on RMST pseudo-value regression. Depressive symptoms mediated 7.2% of the total effect for CVD, 5.9% for heart disease, and 6.8% for stroke. Physical inactivity accounted for 4.5%, 4.6%, and 5.4% of the effects for CVD, heart disease, and stroke, respectively. Current smoking explained 4.5%, 3.3%, and 4.1% of the associations for CVD, heart disease, and stroke, respectively.

**Table 3:** Proportions of the association between SLEs exposure and cardiovascular outcomes attributable to depression, physical inactivity, and current smoking

	Cardiovascular disease			Heart disease			Stroke	
	RMST difference (95% CI), months	PERM, %		RMST difference (95% CI)	PERM, %		RMST difference (95% CI)	PERM, %
**Depressive symptoms**								
No-exposure	0 (reference)			0 (reference)			0 (reference)	
Exposure, multivariate model	-2.22 (-2.90, -1.53)			-1.52 (-2.07, -0.96)			-0.74 (-1.09, -0.39)	
Exposure, depressive symptoms adjusted	-2.06 (-2.75, -1.37)	44.4		-1.43 (-1.99, -0.87)	36.3		-0.69 (-1.05, -0.33)	43.9
**Physical inactivity** ^**¶**^								
No-exposure	0 (reference)			0 (reference)			0 (reference)	
Exposure, multivariate model	-2.22 (-2.90, -1.53)			-1.52 (-2.07, -0.96)			-0.74 (-1.09, -0.39)	
Exposure, physical inactivity adjusted	-2.12 (-2.80, -1.43)	20.2		-1.45 (-2.01, -0.89)	20.0		-0.70 (-1.05, -0.35)	24.8
**Current smoking** ^**¶**^								
No-exposure	0 (reference)			0 (reference)			0 (reference)	
Exposure, multivariate model	-2.22 (-2.90, -1.53)			-1.52 (-2.07, -0.96)			-0.74 (-1.09, -0.39)	
Exposure, current smoking adjusted	-2.12 (-2.81, -1.44)	32.3		-1.47 (-2.02, -0.91)	26.0		-0.71 (-1.06, -0.36)	34.2

Multivariate model was adjusted for age, sex, education, drinking status, body mass index, hypertension, and diabetes

CI, confidence interval; HR, hazard ratio; PERM, percentage of excess risk mediated; SLEs, stressful life events

### Sensitivity analysis and subgroup analysis

The association between adulthood SLE exposure and incident CVD remained robust across multiple sensitivity analyses (Additional file: Tables S6-S11). When using second-wave mediator measurements, the supplementary analysis yielded qualitatively consistent, albeit quantitatively attenuated, mediation effects (Additional file: Table S12). Event-specific analyses revealed that traumatic events exhibited stronger associations with incident CVD compared to non-traumatic or bereavement-related events (Additional file: S13). Results based on a depressive symptom weighted SLE score were largely consistent with those obtained using the simple event count (Additional file: S14). Subgroup analyses demonstrated consistent associations between SLE exposure and CVD risk across age, and sex (Additional file: Tables S15-S16).

## Discussion

This present study provides epidemiological evidence for the association between exposure to SLEs during adulthood and risks of incident CVD, heart disease, and stroke. Through harmonized analysis of two nationally representative aging cohorts (HRS and ELSA), our findings extend current knowledge by demonstrating: (1) exposure to SLEs during adulthood is significantly associated with increased risks of incident CVD, heart disease and stroke, and corresponds to a reduction in event-free survival time, after comprehensive adjustment for traditional risk factors; (2) PAF analyses quantify that 2.6% of cardiovascular events could theoretically be attributed to adulthood SLE exposure; (3) mediation analysis suggest that depression, physical activity, and smoking might mediate the association between exposure to SLEs during adulthood and risk of incident CVD. These findings highlight the potential importance of public health interventions aimed at addressing the impact of SLEs during adulthood, as they may contribute to cardiovascular disease risk. The consistency of these associations across two distinct healthcare systems (US and UK) supports the generalizability of conclusions and suggests the importance of addressing psychosocial stressors in comprehensive CVD prevention strategies.

Current evidence regarding the association between exposure to SLEs during adulthood and CVD risk, primarily derived from case-control studies and a limited number of prospective cohorts, shows considerable heterogeneity in reported findings. A Swedish prospective cohort study involving 4,004 individuals found that financial stress significantly increased the risk of incident CVD, defined as a composite of fatal and nonfatal myocardial infarction, ischemic stroke, and angina-related hospitalization. This effect was observed only in men, particularly among those living without a partner [20]. In contrast, data from the Women’s Health Initiative Observational Study and Clinical Trials cohort, which included 10,785 postmenopausal Black women, indicated no significant association between life event stress and a broad composite CVD outcome over a 12.5-year period in multivariable-adjusted analyses [21].

In addition, existing studies have produced divergent results regarding the associations when coronary heart disease and stroke are evaluated independently. The National Epidemiologic Survey on Alcohol and Related Conditions indicated that the frequency of SLEs experienced in the past year was significantly linked to a composite outcome that included self-reported incidents of arteriosclerosis, angina, or myocardial infarction during a 3-year follow-up of a large, multiethnic cohort of 28,583 U.S. adults [22]. Conversely, in the Copenhagen City Heart Study, which involved 8,738 participants, major life events occurring in childhood, adulthood, and at work were not significantly associated with the incidence of coronary heart disease over a 15-year follow-up period [23]. Additionally, in a large cohort of 12,866 men aged 35 to 57 years at high risk for coronary heart disease, the number of life events reported during each of the 6 years of follow-up showed no relationship to the risk of CHD-related death or fatal and nonfatal myocardial infarction in the subsequent year. However, when angina symptoms were used as a more subjective cardiovascular endpoint, the number of life events emerged as a significant predictor of angina [24]. Regarding stroke as the endpoint, another analysis from the Women’s Health Initiative Observational Study found that life event stress was not significantly linked to incident stroke when adjusted for cardiovascular risk factors [25]. However, findings from the Copenhagen City Heart Study showed that major life events were significantly associated with incident ischemic stroke, even after adjusting for cardiovascular risk factors [23].

The inconsistencies across prior studies on the association between SLEs and CVD risk may largely stem from methodological differences, particularly in how SLEs are operationalized. A common limitation is the aggregation of events with varying severity, chronicity, and psychological impact into a single composite score, which may obscure differential effects on CVD. For example, treating traumatic loss and unemployment as equivalent could mask distinct risk pathways. Differences in study populations and follow-up durations further contribute to heterogeneous findings.

To address these issues, we conducted sensitivity analyses examining SLEs both individually and using a weighted score based on depressive symptom burden. Event-specific analyses indicated that traumatic events (e.g., life-threatening illness) were more strongly associated with CVD outcomes than non-traumatic events (e.g., unemployment). However, analyses employing the weighted SLE score by depressive symptoms produced results that were largely consistent with our primary analyses using the unweighted cumulative count. The limited added predictive value of the weighted score, despite observed event-specific heterogeneity, may reflect that depressive symptoms, while correlated with various SLEs, represent only one dimension of the stress response. This single-anchor approach may not fully capture the distinct physiological, behavioral, and affective mechanisms linking different stressors to CVD risk. For instance, although asset poverty received the highest weight due to its strong cross-sectional association with depressive symptoms, its impact on cardiovascular health may operate through chronic material deprivation rather than via the depressive pathway itself. Moreover, the high correlation between weighted and unweighted scores suggests that weighting did not substantially alter individual rankings of overall stress burden, indicating that the simple cumulative count adequately captured most SLE-related variance in CVD risk in this cohort.

In primary analyses using the unweighted cumulative SLE count, we observed a clear dose-response relationship between SLE exposure and CVD outcomes. After full adjustment for traditional risk factors, each additional SLE was associated with a 1.33-month reduction in event-free survival for CVD, 1.01 months for heart disease, and 0.42 months for stroke over 7.8 years. PAF estimates indicated that 2.6% of incident CVD, 1.7% of heart disease, and 0.8% of stroke cases were attributable to SLE exposure in adulthood. Mediation analyses further identified depressive symptoms, physical inactivity, and current smoking as significant mediators, accounting for 7.2%, 4.5%, and 4.5% of the total effect of SLEs on CVD, respectively.

The findings of this study further emphasize the critical role of psychosocial stressors in the pathogenesis of CVD. Our results closely align with existing research in the field of stress and health, which can be elucidated through several potential mechanisms [26]. First, chronic or severe stress can disrupt cardiovascular equilibrium by affecting the neuroendocrine and autonomic nervous systems. This disruption leads to sustained glucocorticoid secretion from the overactive hypothalamic-pituitary-adrenal axis, promoting inflammation, endothelial dysfunction, and atherosclerosis [27, 28]. Additionally, the release of catecholamines during stress creates an autonomic imbalance, resulting in clinically significant increases in resting heart rate and reductions in heart rate variability, which may precipitate arrhythmias [29]. The observed dose-response relationship between SLEs and CVD risk in this study underscores the cumulative burden of psychosocial stress, a phenomenon consistent with the allostatic load framework. This framework posits that repeated or prolonged activation of stress responses can lead to “wear and tear” on the body, ultimately increasing susceptibility to chronic diseases such as CVD [30]. Second, vascular homeostasis is compromised through both acute and chronic stress mechanisms. Acute psychological stress reduces flow-mediated dilation, while chronic stress upregulates the expression of intercellular adhesion molecule-1 [31]. Concurrently, individuals experiencing stress exhibit increased platelet aggregation and elevated fibrinogen levels, collectively establishing a prothrombotic state [27]. Third, chronic stress induces metabolic dysregulation, characterized by impaired glucose tolerance and elevated blood glucose and lipid levels [32]. Finally, the activation of inflammatory pathways may contribute to the pathogenesis and progression of CVD [33].

Of particular relevance, the mediation analyses in this study provide insights into the pathways through which SLEs may influence cardiovascular health. Depressive symptoms emerged as the most substantial mediator, accounting for 7.2% of the excess risk for incident CVD. This finding aligns with extensive literature documenting the bidirectional relationship between stress, depression, and CVD [34]. Depression is known to exacerbate inflammation, promote platelet aggregation, and reduce heart rate variability, all of which are established risk factors for CVD. Furthermore, depression often leads to poor adherence to medical treatments and unhealthy behaviors, compounding its detrimental effects on cardiovascular health [35]. Physical inactivity and smoking also mediated the relationship between SLEs exposure and CVD risk, albeit to a lesser extent than depression. The negative PAFs for vigorous physical activity suggest that low activity levels significantly contribute to CVD risk among individuals exposed to SLEs. This is consistent with evidence that stress can reduce motivation for physical activity, while inactivity, in turn, exacerbates stress and its physiological consequences [36]. Similarly, smoking, which is often used as a coping mechanism for stress, mediated 4.5% of the excess CVD risk. Nicotine and other toxins in tobacco smoke directly damage the endothelium and promote atherosclerosis, while stress-related smoking may further amplify these effects through synergistic pathways [37].

### Limitations

This study has notable strengths, including its use of large nationally representative cohorts, prospective longitudinal design, and comprehensive covariate adjustment. However, several limitations should be considered. First, this study relies on self-reported measures for both SLEs and CVD, introducing two sources of bias. Recall bias in SLE reporting may overestimate the association, as participants with CVD may recall more stressors. Conversely, non-differential misclassification of self-reported CVD typically attenuates hazard ratios toward the null. The net direction of bias is therefore uncertain. Nevertheless, sensitivity analyses excluding participants with characteristics associated with lower self-report accuracy yielded consistent findings, suggesting that the observed associations are unlikely to be fully driven by reporting error. Future studies using clinically adjudicated or registry-linked endpoints would further improve accuracy. Second, although extensive covariates were adjusted for, residual confounding from unmeasured factors, such as genetic susceptibility, diet, or environmental exposures, cannot be ruled out. Third, the primary analysis employed an unweighted cumulative SLE count, which assumes that each event contributes equally to cardiovascular risk and does not account for differences in the severity, chronicity, or recency of individual events. To address this, we conducted sensitivity analyses using event-specific models and a depressive symptom-weighted score. However, as the weighted score was derived from cross-sectional associations, it may still not fully capture event-specific severity or psychological burden. Fourth, as an observational study, our findings cannot establish definitive causal relationships between SLEs and CVD, despite the temporal sequence suggested by our longitudinal design. Fifth, although mediation analyses identified depression, physical inactivity, and smoking as potential pathways, these findings should be interpreted with caution. While a lagged mediator analysis yielded consistent findings, our primary analysis assessed mediators only at baseline and did not fully capture their time-varying nature. Moreover, participants with baseline depression, smoking, or physical inactivity were not excluded, and lifetime histories of these mediators were not accounted for, which may introduce residual bias. Future studies incorporating repeated measurements of mediators over time and accounting for lifetime histories are warranted to more precisely quantify these indirect pathways. Finally, the generalizability of our results may be limited to older adults (aged ≥ 50 years) in high-income, Western settings such as the U.S. and the U.K. Our findings may not extend to younger populations, who face different developmental stressors and exhibit distinct physiological responses to stress than older adults. Furthermore, the applicability of our findings to other racial, ethnic, and socioeconomic contexts may be limited, which is particularly salient for stroke given well-documented disparities in its epidemiology. For instance, a recent ecological study found that East Asians exhibited significantly higher incidence of both ischemic and hemorrhagic stroke than Caucasians [38]. These pronounced differences in stroke subtype distribution suggest that the pathways linking SLEs to CVD risk may operate differently across racial groups.

## Conclusions

This study provides evidence that exposure to SLEs during adulthood is associated with increased CVD risk, independent of traditional factors. Using RMST analysis, we observed a clear dose-response relationship between SLE exposure and reduced cardiovascular event-free survival. Our findings suggest that this association may involve multiple pathways, including depression, physical inactivity, and smoking. These results highlight the importance of addressing psychosocial stress in cardiovascular prevention strategies, both through clinical interventions targeting stress management and behavioral risk factors, as well as broader public health initiatives.

## Supplementary Information

Below is the link to the electronic supplementary material.

Supplementary Material 1: Supplemental Material: Construction of the Weighted Stressful Life Events Score and Reanalysis

Supplementary Material 2: Additional file 1: Tables S1–S16 and Fig. S1.

## Data Availability

The datasets used in this study are available after approval of an application from the following sources: Health and Retirement Study: https://hrsonline.isr.umich.edu; English Longitudinal Study of Ageing: https://ifs.org.uk/ELSA.

## Declarations

### Clinical Trial

Not available.

### Authors’ social media handles

Gregory Y. H. Lip – X: @LiverpoolCCS.

## Abbreviations

BMI: Body mass index
CES-D-8: 8-item Center for Epidemiologic Studies Depression Scale
CI: Confidence interval
CVD: Cardiovascular disease
ELSA: English Longitudinal Study on Ageing
HR: Hazard ratio
HRS: Health and Retirement Study
PAF: Population attributable fraction
PERM: Percentage of excess risk mediated
RMST: Restricted mean survival time
SLE: Stressful life event
