# Supplementary material for: Adulthood stressful life events as predictors of incident cardiovascular disease: insights from two prospective cohorts
Source: BMC Med. 2026 Apr 24;24:349. doi: 10.1186/s12916-026-04890-0 (PMC13244983; doi:10.1186/s12916-026-04890-0)
Supplement: Supplementary file 1 — Supplementary Material 1: Supplemental Material: Construction of the Weighted Stressful Life Events Score and Reanalysis [file 12916_2026_4890_MOESM1_ESM.pdf]

Supplemental Material: Construction of the Weighted Stressful Life Events Score and Reanalysis

To address the reviewer's concern regarding the potential heterogeneity in the cardiovascular impact of different stressful life events (SLEs), we constructed an alternative weighted SLE score. This score aims to reflect the varying degrees of psychological impact associated with different types of life events, moving beyond the assumption that all events contribute equally to cumulative stress burden. The weighting scheme was derived empirically from our own data, based on each event's association with concurrent depressive symptoms, which is a well-established proximal outcome of psychological stress.

Details of the analytical method are provided below:

1. Variables

1.1 Dependent Variable (Anchor for Weighting)
- CES-D Total Score: A continuous measure of depressive symptomatology (range typically 0-8 in our harmonized measure), with higher scores indicating more severe depressive symptoms.

1.2 Independent Variables (Stressful Life Events)
All events were coded as binary indicators (1 = event occurred; 0 = event did not occur):
1. work: Unemployment (vs. employment)
2. illness: Experienced a life-threatening illness or accident
3. attack: Experienced a physical attack or injury
4. widowed: Death of a spouse or partner
5. asset: Asset poverty (vs. asset sufficiency)
6. child: Death of a child

2. Statistical Method for Weight Derivation

We estimated the relative psychological impact of each SLE by fitting a simple linear regression model:

$$\text{CES-D} = \beta0 + \beta1 \times \text{work} + \beta2 \times \text{illness} + \beta3 \times \text{attack} + \beta4 \times \text{widowed} + \beta5 \times \text{asset} + \beta6 \times \text{child} + \varepsilon$$

No additional covariates (e.g., age, sex, education) were included in this model to avoid over-adjustment, as these factors would later be controlled for in the primary Cox proportional hazards models. The model was fitted using ordinary least squares.

3. Model Results and Coefficient Estimates

The regression model summary is presented below:

Predictor	Description	Estimate (β)	Std. Error	t value	Pr(>|t|)
(Intercept)		1.09012	0.01682	64.815	$< 2.2 \times 10^{-16}$
work	Unemployment	0.50925	0.07959	6.398	1.61×10^{-10}
illness	Life-threatening illness	0.21971	0.04247	5.174	2.32×10^{-7}
attack	Physical attack	0.61606	0.07388	8.338	$< 2.2 \times 10^{-16}$
widowed	Death of spouse	0.57439	0.03826	15.011	$< 2.2 \times 10^{-16}$
asset	Asset poverty	0.99388	0.04084	24.337	$< 2.2 \times 10^{-16}$
child	Death of child	0.08861	0.06163	1.438	0.1505

Model Diagnostics:
Residual standard error: 1.864 on 18,891 degrees of freedom
Multiple R-squared: 0.05116, Adjusted R-squared: 0.05085

F-statistic: 169.7 on 6 and 18,891 DF, p-value: $< 2.2\times10^{-16}$

4. Weight Calculation Procedure

The absolute values of the regression coefficients (β) were used as the basis for weight calculation, reflecting the magnitude of association with depressive symptoms regardless of direction.

Step 1: Identify the smallest non-zero absolute coefficient
min(|β| > 0) = |β_child| = 0.08861

Step 2: Calculate raw weights
Each event's absolute coefficient was divided by the minimum non-zero coefficient:
Raw Weight_i = |β_i| / 0.08861

Step 3: Round weights to one decimal place for practical use
Decimal Weight_i = round(Raw Weight_i, 1)

5. Final Weight Assignment

The resulting weights for each stressful life event are as follows:

Stressful Life Event	Beta Estimate	Absolute Beta	Raw Weight	Decimal Weight
Unemployment (work)	0.5093	0.5093	5.747	5.7
Life-threatening illness (illness)	0.2197	0.2197	2.48	2.5
Physical attack (attack)	0.6161	0.6161	6.952	7
Death of spouse (widowed)	0.5744	0.5744	6.482	6.5
Asset poverty (asset)	0.9939	0.9939	11.216	11.2
Death of child (child)	0.0886	0.0886	1	1

Note: The death of a child received a weight of 1.0 as the reference event with the smallest non-zero association with depressive symptoms.

6. Calculation of Individual Weighted SLE Scores

For each participant j, the weighted SLE score was computed as:

Weighted SLE Score_j = Σ (Event_ij × Weight_i)

Where:
- Event_ij is an indicator variable (0 or 1) for whether participant j experienced event i
- Weight_i is the decimal weight assigned to event i as shown in the table above

7. Correlation Analysis Between Weighted and Unweighted Scores

7.1 Score Calculation
Unweighted SLE Score = Σ Event_i (simple count, range: 0-6)
Weighted SLE Score = Σ (Event_i × Weight_i) (continuous score)

7.2 Correlation Results
Pearson correlation analysis between unweighted and weighted SLE scores:
Correlation coefficient (r) = 0.894
95% Confidence Interval: [0.891, 0.897], p-value < 2.2×10^{-16}

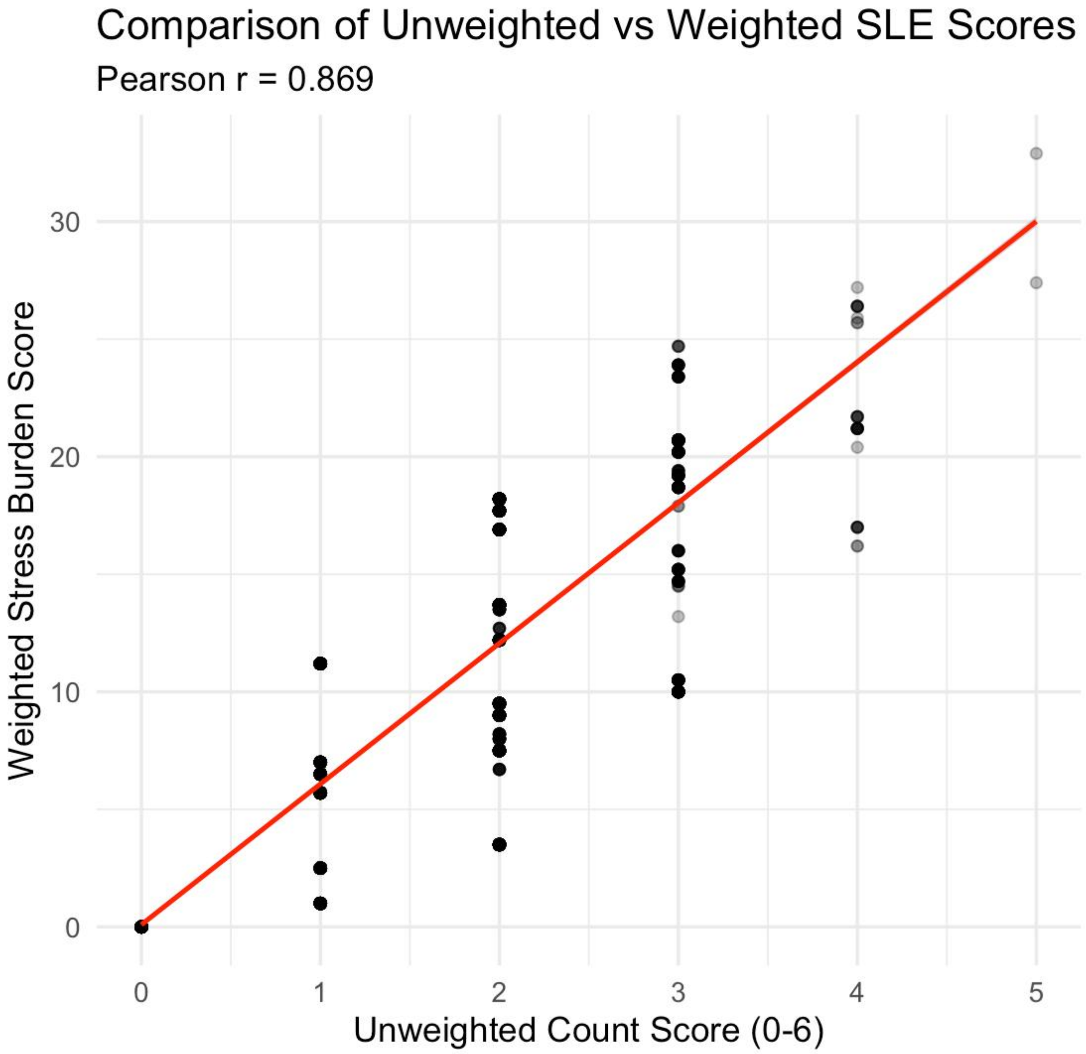

8. Analytical Application

We further evaluated the weighted SLE score as an alternative exposure measure for the association between cumulative stress burden and incident cardiovascular outcomes. The weighted SLE score was analyzed both as a continuous variable and as a categorical variable. The categorical classification was based on traumatic event equivalents, with the death of a spouse (assigned weight = 6.5) defined as one standard traumatic unit. Participants were initially classified into four groups: (1) no events (score = 0); (2) low burden (<1 traumatic equivalent, score >0 to <6.5); (3) moderate burden (1–2 equivalents, score 6.5 to <13); and (4) high burden (≥2 equivalents, score ≥13).

All RMST analysis and Cox proportional hazards models incorporating the weighted SLE score were adjusted for age, sex, education, smoking status, alcohol consumption, body mass index, hypertension, and diabetes. The results are presented below.

Association of weighted stressful life event score with incident cardiovascular outcomes

All participants	Cardiovascular disease			Heart disease			Stroke		
	RMST difference (95% CI)	Cox models		RMST difference (95% CI)	Cox models		RMST difference (95% CI)	Cox models	
		HR (95% CI)	P value		HR (95% CI)	P value		HR (95% CI)	P value
**Model 1**									
**Continuous analysis**									
Per 1-score increase	-0.17 (-0.24, -0.09)	1.01 (1.01, 1.02)	< 0.001	-0.11 (-0.17, -0.06)	1.01 (1.00, 1.02)	0.009	-0.05 (-0.09, -0.01)	1.02 (1.01, 1.03)	0.007
Per 1-traumatic equivalent	-1.00 (-1.44, -0.56)	1.09 (1.04, 1.14)	< 0.001	-0.73 (-1.11, -0.36)	1.07 (1.02, 1.13)	0.009	-0.32 (-0.56, -0.08)	1.13 (1.03, 1.23)	0.007
**Categorical analysis**									
No events	1 (reference)	1 (reference)		1 (reference)	1 (reference)		1 (reference)	1 (reference)	
Low burden	-3.37 (-4.41, -2.32)	1.27 (1.14, 1.42)	< 0.001	-2.36 (-3.25, -1.47)	1.22 (1.07, 1.38)	0.002	-1.48 (-2.05, -0.91)	1.44 (1.17, 1.76)	< 0.001
Moderate-high burden	-2.80 (-3.56, -2.05)	1.31 (1.21, 1.43)	< 0.001	-1.92 (-2.56, -1.28)	1.25 (1.14, 1.37)	< 0.001	-1.04 (-1.46, -0.63)	1.50 (1.28, 1.74)	< 0.001
**Model 2**									
**Continuous analysis**									
Per 1-score increase	-0.14 (-0.21, -0.06)	1.01 (1.00, 1.02)	0.006	-0.10 (-0.16, -0.04)	1.01 (1.00, 1.02)	0.032	-0.03 (-0.07, 0.01)	1.01 (1.00, 1.02)	0.208
Per 1-traumatic equivalent	-0.82 (-1.27, -0.37)	1.07 (1.02, 1.13)	0.006	-0.63 (-1.01, -0.24)	1.06 (1.01, 1.12)	0.032	-0.19 (-0.44, 0.05)	1.06 (0.97, 1.17)	0.208
**Categorical analysis**									
No events	1 (reference)	1 (reference)		1 (reference)	1 (reference)		1 (reference)	1 (reference)	
Low burden	-3.11 (-4.14, -2.07)	1.27 (1.14, 1.42)	< 0.001	-2.16 (-3.04, -1.28)	1.22 (1.07, 1.38)	0.002	-1.35 (-1.91, -0.78)	1.42 (1.16, 1.75)	< 0.001
Moderate-high burden	-1.80 (-2.57, -1.02)	1.17 (1.07, 1.28)	< 0.001	-1.30 (-1.96, -0.64)	1.14 (1.03, 1.26)	0.008	-0.53 (-0.95, -0.10)	1.19 (1.01, 1.40)	0.035

Model 1 was unadjusted.

Model 2 was adjusted for age, sex, education, current drinking, body mass index, hypertension, and diabetes.

CI, confidence interval; ELSA, English Longitudinal Study of Ageing; HR, hazard ratio; HRS, Health and Retirement Study; RMST, restricted mean survival time.
