# Supplementary material for: Adulthood stressful life events as predictors of incident cardiovascular disease: insights from two prospective cohorts
Source: BMC Med. 2026 Apr 24;24:349. doi: 10.1186/s12916-026-04890-0 (PMC13244983; doi:10.1186/s12916-026-04890-0)
Supplement: Supplementary file 2 — Supplementary Material 2: Additional file 1: Tables S1–S16 and Fig. S1. [file 12916_2026_4890_MOESM2_ESM.docx]

**Supplemental Table Content**

Supplementary Table S1. Definition and cross-cohort harmonization of adulthood stressful life event indicators in HRS and ELSA

Supplementary Table S2. Definitions and harmonization of variables across HRS and ELSA cohorts

Supplementary Table S3. Missing data (counts and rates) for covariates in the HRS and ELSA cohorts

Supplementary Table S4. Characteristics of participants in each cohort stratified by adulthood stressful life events exposure

Supplementary Table S5. Association of adulthood stressful life events exposure with risks of incident cardiovascular diseases with analyses stratified by cohort

Supplementary Table S6. Association of adulthood stressful life events exposure with risks of incident cardiovascular diseases using data not being imputed

Supplementary Table S7. Association of adulthood stressful life events exposure with risks of incident cardiovascular diseases with additional adjustment for antihypertensive and antidiabetic medications

Supplementary Table S8. Association of adulthood stressful life events exposure with risks of incident cardiovascular diseases using the competing risk model

Supplementary Table S9. Association of adulthood stressful life events exposure with risks of incident cardiovascular diseases at different follow-up time-points

Supplementary Table S10. Association of adulthood stressful life events exposure with risks of incident cardiovascular diseases excluding participants who were diagnosed with CVD within the first 2 years of follow-up

Supplementary Table S11. Association of adulthood stressful life events exposure with risks of incident cardiovascular diseases excluding participants with low related to self-report accuracy

Supplementary Table S12. Mediation analysis employing second-wave measurements

Supplementary Table S13. Association of each adulthood stressful life event component with incident cardiovascular outcomes

Supplementary Table S14. Association of depression-weighted stressful life event score with incident cardiovascular outcomes

Supplementary Table S15. Association of adulthood stressful life events exposure with risks of incident cardiovascular diseases stratified by age

Supplementary Table S16. Association of adulthood stressful life events exposure with risks of incident cardiovascular diseases stratified by sex

Supplementary Figure S1. Flow chart of participant selection

**Supplementary Table S1. Definition and cross-cohort harmonization of adulthood stressful life event indicators in HRS and ELSA**

| **Indicators** | **Cohort** | **Source Variable Name & Description** | **Original Values / Codes** | **Harmonization Rule & Final Analytic Variable** |
| --- | --- | --- | --- | --- |
| Unemployment | HRS | NJ005M1: Current job status | 2=Unemployed and looking for work, 4=Disabled, 5=Retired, 6=Homemaker, 7=Other, 8=On leave, 1=Working, 3=Laid off | Present = 1: HRS status=2 or ELSA status=4 (“unemployed”) code as 1  Absent = 0: All other categories coded as 0 |
|  | ELSA | wselfd: Self-reported employment status | 4=unemployed, 3=retired, 5=lt_sick, 6=other, 1=employee, 2=self_emp |  |
| Asset Poverty | HRS | H11ATOTB: Total of all assets (cross-wave) | Continuous | Present = 1: Total net wealth ≤ 0 code as 1  Absent = 0: Total net wealth > 0 code as 0 |
|  | ELSA | netfw_bu_s: BU total net financial wealth | Continuous |  |
| Death of Child | HRS | NLB037A: Has a child of yours ever died | 1=Yes, 5=No | Present = 1: HRS response=1 or ELSA has non-missing year of child’s death (rcdyy/rcndy) code as 1  Absent = 0: HRS response=5 or ELSA has missing year of child’s death (rcdyy/rcndy) code as 0 |
|  | ELSA | rcdyy: Year of child’s death; rcndy: Other natural child’s year of death | Year or missing |  |
| Death of Spouse/Partner | HRS | Derived from marital status (R11MSTAT) | 1=Married, 2=Married spouse absent, 3=Partnered, 4=Separated, 5=Divorced, 6=Sep/Div, 7=Widowed, 8=Never married | Present = 1: HRS status=7 (“Widowed”) or ELSA status=7 (“Widowed”) or 10 (“Surviving civil partner”) code as 1  Absent = 0: All other categories coded as 0 |
|  | ELSA | Derived from marital status (dimar) | 2=Married (1st), 3=Civil partner, 4=Remarried, 11=Civil partner (remarried), 1=Single, 5=Legally separated, 6=Divorced, 7=Widowed, 8=Civil partner separated, 9=Civil partnership dissolved, 10=Surviving civil partner |  |
| Life-Threatening Illness | HRS | NLB037F: Ever had a life-threatening illness or accident | 1=Yes, 5=No | Present = 1 (HRS:1; ELSA:1) vs. Absent = 0 (HRS:5; ELSA:2) |
|  | ELSA | rsill: Ever had a life-threatening illness or accident | 1=Yes, 2=No |  |
| Physical Attack | HRS | NLB037E: Ever victim of physical attack | 1=Yes, 5=No | Present = 1 (HRS:1; ELSA:1) vs. Absent = 0 (HRS:5; ELSA:2) |
|  | ELSA | rsattac: Ever been a victim of serious physical attack | 1=Yes, 2=No |  |

**Supplementary Table S2. Definitions and harmonization of variables across HRS and ELSA cohorts**

| **Variable** | **Cohort** | **Source Variable Name & Description** | **Original Values / Codes** | **Harmonization Rule & Final Analytic Variable** |
| --- | --- | --- | --- | --- |
| Age | HRS | R11AGEY_E: Age at interview end (years) | Continuous | Continuous (years) |
|  | ELSA | dhager: Age collapsed / INDAGER: Age in years | Continuous |  |
| Sex | HRS | RAGENDER: Gender | 1=Male, 2=Female | Binary: 1=Male, 2=Female |
|  | ELSA | disex: Respondent sex | 1=Male, 2=Female |  |
| Educational Attainment | HRS | RAEDUC: Education (categorical) | 1=Lt High-school, 2=GED, 3=High-school graduate, 4=Some college, 5=College and above | Three categories: 1. Below high school (HRS:1; ELSA:7). 2. High school (HRS:2,3; ELSA:4,5). 3. College or above (HRS:4,5; ELSA:1,2,3) |
|  | ELSA | edqual: Educational qualification – merged | 1=nvq4/nvq5/degree, 2=higher ed below degree, 3=nvq3/gce a level, 4=nvq2/gce o level, 5=nvq1/cse other, 6=foreign/other, 7=no qualification |  |
| Marital Status | HRS | R11MSTAT: Marital Status | 1=Married, 2=Married spouse absent, 3=Partnered, 4=Separated, 5=Divorced, 6=Sep/Div, 7=Widowed, 8=Never married | Binary: Married/Partnered (HRS:1,2,3; ELSA:2,3,4,11) vs. Other (all other categories) |
|  | ELSA | dimar: Current legal marital status | 2=Married (1st), 3=Civil partner, 4=Remarried, 11=Civil partner (remarried), 1=Single, 5=Legally separated, 6=Divorced, 7=Widowed, 8=Civil partner separated, 9=Civil partnership dissolved, 10=Surviving civil partner |  |
| Current Smoking | HRS | R11SMOKEN: R smokes now | 1=Yes, 5=No | Binary: Current smoker (HRS:1; ELSA:1) vs. Non-smoker (HRS:5; ELSA:0) |
|  | ELSA | smoker: Whether current smoker | 1=current smoker, 0=not a current smoker |  |
| Current Drinking | HRS | R11DRINKN: R # drinks/day when drinks | Drinks/day when drinks | Binary: Current drinker (HRS:≥1; ELSA:1-7) vs. Non-drinker (HRS:0; ELSA:8) |
|  | ELSA | scako: How often had alcoholic drink last 12 months | 1=Almost every day, 2=5-6 days/wk, 3=3-4 days/wk, 4=1-2 days/wk, 5=1-2 days/mo, 6=Every couple months, 7=1-2 days/yr, 8=Not at all |  |
| Physical Activity | HRS | R11VGACTX: Frequency of vigorous physical activity; R11MDACTX: Frequency of moderate physical activity; R11LTACTX: Frequency of light physical activity | 1=More than once/wk, 2=Once/wk, 3=1-3 times/mo, 4=Hardly ever, 5=Never | Three levels: Vigorous: Any vigorous activity (HRS: R11VGACTX in 1-3; ELSA: heacta in 1-3). Moderate: No vigorous activity, but any moderate activity (HRS: R11MDACTX in 1-3; ELSA: heactb in 1-3). Light: No vigorous & no moderate activity, but any mild activity (HRS: R11LTACTX in 1-3; ELSA: heactc in 1-3) |
|  | ELSA | heacta: Frequency of vigorous sports/activities; heactb: Frequency of moderate sports/activities; heactc: Frequency of mild sports/activities | -9=Refusal, -8=Don’t know, -2=Schedule not applicable, -1=Item not applicable, 1=More than once/wk, 2=Once/wk, 3=1-3 times/mo, 4=Hardly ever/never |  |
| Body Mass Index (BMI) | HRS | R11BMI: Self-reported BMI (kg/m²) | Continuous | Continuous (kg/m²) |
|  | ELSA | bmival: BMI calculated from measured height and weight | Continuous |  |
| Depressive Symptoms | HRS | R11CESD: CES-D-8 total score | 0-8 | Total score (0-8) |
|  | ELSA | totalpsc: CES-D-8 total score | 0-8 |  |
| Hypertension | HRS | R11HIBPE: R ever had high blood pressure | 1=Yes, 5=No | Binary: Yes (HRS:1; ELSA:1) vs. No (HRS:5; ELSA:2) |
|  | ELSA | hedawbp: Diagnosed high blood pressure (fed forward) | 1=Yes, 2=No |  |
| Diabetes | HRS | R11DIAB: R reports diabetes this wave | 1=Yes, 5=No | Binary: Yes (HRS:1; ELSA:1) vs. No (HRS:5; ELSA:2) |
|  | ELSA | hedawdi: Diagnosed diabetes or high blood sugar (fed forward) | 1=Yes, 2=No |  |
| Anti-hypertensive Medication | HRS | NC006: Blood pressure medication now taking | 1=Yes, 5=No | Binary: Yes (HRS:1; ELSA:1) vs. No (HRS:5; ELSA:2) |
|  | ELSA | Hemda: High blood pressure: whether taking medication | 1=Yes, 2=No |  |
| Anti-diabetic Medication | HRS | NC011: Swallowed medication for diabetes | 1=Yes, 5=No | Binary: Yes (HRS:1; ELSA:1) vs. No (HRS:5; ELSA:2) |
|  | ELSA | HeMdb: Diabetes: whether taking medication | 1=Yes, 2=No |  |
| Incident heart problem | HRS | RxHEARTS: R had heart problem since last interview | 0=No, 1=Yes | Binary: Incident Case: First report of “Yes” (1) in HRS and first report of “Mentioned” (1) in ELSA. Absence of Event: No such report (HRS) or no such change (ELSA) during follow-up (censored). |
|  | ELSA | hediaan: angina diagnosis newly reported; hediami: heart attack diagnosis newly reported;hediahf: congestive heart failure diagnosis newly reported;  hedia95: other heart disease diagnosis newly reported | -9.0 = Refusal,  -8.0 = Don't Know,  -2.0 = Schedule not applicable,  -1.0 = Item not applicable,  0.0 = Not mentioned,  1.0 = Mentioned |  |
| Incident Stroke | HRS | RxSTROKS: R had stroke since last interview | 0=No, 1=Yes | Binary: Incident Case: First report of “Yes” (1) in HRS and first report of “Mentioned” (1) in ELSA. Absence of Event: No such report (HRS) or no such change (ELSA) during follow-up (censored). |
|  | ELSA | hediast: stroke diagnosis newly reported | -9.0 = Refusal,  -8.0 = Don't Know,  -2.0 = Schedule not applicable,  -1.0 = Item not applicable,  0.0 = Not mentioned,  1.0 = Mentioned |  |

**Supplementary Table S3. Missing data (counts and rates) for covariates in the HRS and ELSA cohorts**

| **Variables** | **HRS** | | | |  | **ELSA** | | | |
| --- | --- | --- | --- | --- | --- | --- | --- | --- | --- |
|  | **Total (n=13399)** | **No SLEs (n=8349)** | **1-2 SLEs (n=4840)** | **> 2 SLEs  (n=177)** |  | **Total (n=5532)** | **No SLEs (n=2866)** | **1-2 SLEs (n=2556)** | **> 2 SLEs  (n=110)** |
| **Age** | 0(0.00%) | 0(0.00%) | 0(0.00%) | 0(0.00%) |  | 0(0.00%) | 0(0.00%) | 0(0.00%) | 0(0.00%) |
| **Sex** | 0(0.00%) | 0(0.00%) | 0(0.00%) | 0(0.00%) |  | 0(0.00%) | 0(0.00%) | 0(0.00%) | 0(0.00%) |
| **Education** | 3(0.02%) | 2(0.02%) | 1(0.02%) | 0(0.00%) |  | 448(8.10%) | 214(7.47%) | 223(8.72%) | 11(10.00%) |
| **Marital status** | 0(0.00%) | 0(0.00%) | 0(0.00%) | 0(0.00%) |  | 0(0.00%) | 0(0.00%) | 0(0.00%) | 0(0.00%) |
| **Current smoking** | 76(0.57%) | 45(0.54%) | 30(0.62%) | 1(0.56%) |  | 0(0.00%) | 0(0.00%) | 0(0.00%) | 0(0.00%) |
| **Current drinking** | 3(0.02%) | 3(0.04%) | 0(0.00%) | 0(0.00%) |  | 508(9.18%) | 233(8.13%) | 257(10.05%) | 18(16.36%) |
| **Physical activity** | 51(0.38%) | 30(0.36%) | 21(0.43%) | 0(0.00%) |  | 0(0.00%) | 0(0.00%) | 0(0.00%) | 0(0.00%) |
| **BMI** | 232(1.74%) | 115(1.38%) | 113(2.33%) | 4(2.26%) |  | 1766(31.92%) | 907(31.65%) | 813(31.81%) | 46(41.82%) |
| **CESD** | 395(2.96) | 237(2.84%) | 156(3.22%) | 2(1.13%) |  | 12(0.22%) | 7(0.24%) | 5(0.20%) | 0(0.00%) |
| **Hypertension** | 21 (0.16%) | 13(0.16%) | 8(0.17%) | 0(0.00%) |  | 0(0.00%) | 0(0.00%) | 0(0.00%) | 0(0.00%) |
| **Diabetes** | 11(0.08%) | 6(0.07%) | 4(0.08%) | 1(0.56%) |  | 0(0.00%) | 0(0.00%) | 0(0.00%) | 0(0.00%) |
| **Antihypertensive drugs** | 21 (0.16%) | 13(0.16%) | 8(0.17%) | 0(0.00%) |  | 1(0.02) | 0(0.00%) | 1(0.04%) | 0(0.00%) |
| **Antidiabetic drugs** | 11(0.08%) | 6(0.07%) | 4(0.08%) | 0(0.00%) |  | 0(0.00%) | 0(0.00%) | 0(0.00%) | 0(0.00%) |

BMI, body mass index; CESD, Center of Epidemiologic Studies Depression Scale; ELSA, English Longitudinal Study of Ageing; HRS, Health and Retirement Study; SLEs, stressful life events

**Supplementary Table S4. Characteristics of participants in each cohort stratified by adulthood stressful life events exposure**

| **Variables** | **HRS** | | | | |  | **ELSA** | | | | |
| --- | --- | --- | --- | --- | --- | --- | --- | --- | --- | --- | --- |
|  | **Total (n=13399)** | **No SLEs (n=8349)** | **1-2 SLEs (n=4840)** | **> 2 SLEs  (n=177)** | **P value** |  | **Total (n=5532)** | **No SLEs (n=2866)** | **1-2 SLEs (n=2556)** | **> 2 SLEs  (n=110)** | **P value** |
| **Age, mean (SD), years** | 64.98 ± 11.02 | 63.83 ± 9.96 | 66.98 ± 12.35 | 64.34 ± 12.43 | <0.001 |  | 63.41 ± 10.51 | 62.14 ± 9.58 | 64.80 ± 11.24 | 64.33 ± 11.75 | <0.0001 |
| **Sex, n (%)** |  |  |  |  | <0.001 |  |  |  |  |  | 0.12 |
| **Male** | 5191(38.84) | 3603(43.15) | 1537(31.76) | 51(28.81) |  |  | 2334(42.19) | 1244(43.41) | 1049(41.04) | 41(37.27) |  |
| **Female** | 8175(61.16) | 4746(56.85) | 3303(68.24) | 126(71.19) |  |  | 3198(57.81) | 1622(56.59) | 1507(58.96) | 69(62.73) |  |
| **Education, n (%)** |  |  |  |  | <0.001 |  |  |  |  |  | <0.0001 |
| **Below high school** | 2371(17.74) | 1124(13.46) | 1197(24.73) | 50(28.25) |  |  | 1773(32.05) | 782(27.29) | 938(36.70) | 53(48.18) |  |
| **High school** | 4396(32.89) | 2686(32.17) | 1658(34.26) | 52(29.38) |  |  | 2752(49.75) | 1481(51.67) | 1227(48.00) | 44(40.00) |  |
| **College or above** | 6599(49.37) | 4539(54.37) | 1985(41.01) | 75(42.37) |  |  | 1007(18.20) | 603(21.04) | 391(15.30) | 13(11.82) |  |
| **Marital status, n (%)** |  |  |  |  | <0.001 |  |  |  |  |  | <0.0001 |
| **Married or partnered** | 8778(65.67) | 6778(81.18) | 1949(40.27) | 51(28.81) |  |  | 3897(70.44) | 2400(83.74) | 1467(57.39) | 30(27.27) |  |
| **Other marital status** | 4588(34.33) | 1571(18.82) | 2891(59.73) | 126(71.19) |  |  | 1635(29.56) | 466(16.26) | 1089(42.61) | 80(72.73) |  |
| **Current smoking, n (%)** | 1995(14.93) | 1105(13.24) | 842(17.40) | 48(27.12) | <0.001 |  | 777(14.05) | 337(11.76) | 410(16.04) | 30(27.27) | <0.0001 |
| **Current drinking, n (%)** | 7692(57.55) | 5110(61.20) | 2490(51.45) | 92(51.98) | <0.001 |  | 4974(89.91) | 2659(92.78) | 2232(87.32) | 83(75.45) | <0.0001 |
| **Physical activity, n (%)** |  |  |  |  | <0.001 |  |  |  |  |  | <0.0001 |
| **Light level** | 2175(16.27) | 1063(12.73) | 1066(22.02) | 46(25.99) |  |  | 645(11.66) | 237( 8.27) | 381(14.91) | 27(24.55) |  |
| **Moderate level** | 4550(34.04) | 2744(32.87) | 1743(36.01) | 63(35.59) |  |  | 2521(45.57) | 1273(44.42) | 1187(46.44) | 61(55.45) |  |
| **Vigorous level** | 6641(49.69) | 4542(54.40) | 2031(41.96) | 68(38.42) |  |  | 2366(42.77) | 1356(47.31) | 988(38.65) | 22(20.00) |  |
| **BMI, mean (SD), kg/m2** | 28.49 ± 6.15 | 28.41 ± 5.89 | 28.58 ± 6.52 | 29.48 ± 7.22 | 0.032 |  | 28.06 ± 4.98 | 27.61 ± 4.59 | 28.51 ± 5.27 | 29.55 ± 6.29 | <0.0001 |
| **CESD, mean (SD)** | 1.38 ± 1.94 | 1.12 ± 1.73 | 1.79 ± 2.17 | 2.29 ± 2.31 | <0.001 |  | 1.35 ± 1.85 | 1.00 ± 1.50 | 1.67 ± 2.05 | 3.16 ± 2.60 | <0.0001 |
| **Hypertension, n (%)** | 7294(54.57) | 4326(51.81) | 2855(58.99) | 113(63.84) | <0.001 |  | 1583(28.62) | 772(26.94) | 779(30.48) | 32(29.09) | 0.02 |
| **Diabetes, n (%)** | 2707(20.25) | 1574(18.85) | 1088(22.48) | 45(25.42) | <0.001 |  | 287( 5.19) | 127( 4.43) | 153( 5.99) | 7( 6.36) | 0.03 |
| **Antihypertensive drugs, n (%)** | 6320(47.28) | 3768(45.13) | 2462(50.87) | 90(50.85) | <0.001 |  | 1442(26.07) | 670(23.38) | 738(28.87) | 34(30.91) | <0.0001 |
| **Antidiabetic drugs, n (%)** | 2135(15.97) | 1257(15.06) | 842(17.40) | 36(20.34) | <0.001 |  | 292( 5.28) | 118( 4.12) | 165( 6.46) | 9( 8.18) | <0.001 |

BMI, body mass index; CESD, Center for Epidemiologic Studies Depression Scale; ELSA, English Longitudinal Study of Ageing; HRS, Health and Retirement Study; SD, Standard Deviation; SLEs, stressful life events.

**Supplementary Table S5. Association of adulthood stressful life events exposure with risks of incident cardiovascular diseases with analyses stratified by cohort**

|  | **Cardiovascular disease** | | | |  | **Heart disease** | | | |  | **Stroke** | | | |
| --- | --- | --- | --- | --- | --- | --- | --- | --- | --- | --- | --- | --- | --- | --- |
|  | **Event rate (events/100 person years)** | **RMST difference**  **(95% CI)** | **Cox models** | |  | **Event rate (events/100 person years)** | **RMST difference**  **(95% CI)** | **Cox models** | |  | **Event rate (events/100 person years)** | **RMST difference**  **(95% CI)** | **Cox models** | |
|  |  |  | **HR (95% CI)** | **P value** |  |  |  | **HR (95% CI)** | **P value** |  |  |  | **HR (95% CI)** | **P value** |
| **HRS** |  |  |  |  |  |  |  |  |  |  |  |  |  |  |
| **Model 1** |  |  |  |  |  |  |  |  |  |  |  |  |  |  |
| Binary SLEs |  |  |  |  |  |  |  |  |  |  |  |  |  |  |
| No SLEs | 2.52 | 0 (reference) | 1 (reference) |  |  | 2.11 | 0 (reference) | 1 (reference) |  |  | 0.65 | 0 (reference) | 1 (reference) |  |
| SLEs | 3.19 | -2.23 (-2.99, -1.46) | 1.28 (1.17, 1.39) | < 0.001 |  | 2.56 | -1.30 (-1.93, -0.66) | 1.20 (1.09, 1.32) | < 0.001 |  | 0.94 | -0.82 (-1.20, -0.44) | 1.45 (1.23, 1.71) | < 0.001 |
| Categorical SLEs |  |  |  |  |  |  |  |  |  |  |  |  |  |  |
| No SLEs | 2.52 | 0 (reference) | 1 (reference) |  |  | 2.11 | 0 (reference) | 1 (reference) |  |  | 0.65 | 0 (reference) | 1 (reference) |  |
| 1-2 SLEs | 3.18 | -2.15 (-2.93, -1.38) | 1.27 (1.16, 1.39) | < 0.001 |  | 2.55 | -1.23 (-1.87, -0.59) | 1.19 (1.08, 1.32) | < 0.001 |  | 0.95 | -0.81 (-1.19, -0.42) | 1.45 (1.23, 1.71) | < 0.001 |
| SLEs > 2 | 3.51 | -4.27 (-7.53, -1.01) | 1.46 (1.03, 2.08) | 0.034 |  | 2.85 | -3.25 (-5.94, -0.56) | 1.27 (0.86, 1.88) | 0.228 |  | 0.88 | -1.25 (-2.86, 0.36) | 1.33 (0.66, 2.68) | 0.430 |
| Continuous SLEs |  |  |  |  |  |  |  |  |  |  |  |  |  |  |
| Per additional SLE | 2.77 | -1.44 (-1.98, -0.90) | 1.17 (1.10, 1.24) | < 0.001 |  | 2.28 | -0.94 (-1.39, -0.50) | 1.14 (1.07, 1.21) | < 0.001 |  | 0.76 | -0.45 (-0.71, -0.18) | 1.22 (1.10, 1.36) | < 0.001 |
| **Model 2** |  |  |  |  |  |  |  |  |  |  |  |  |  |  |
| Binary SLEs |  |  |  |  |  |  |  |  |  |  |  |  |  |  |
| No SLEs | 2.52 | 0 (reference) | 1 (reference) |  |  | 2.11 | 0 (reference) | 1 (reference) |  |  | 0.65 | 0 (reference) | 1 (reference) |  |
| SLEs | 3.19 | -1.45 (-2.23, -0.67) | 1.16 (1.06, 1.27) | 0.001 |  | 2.56 | -0.80 (-1.44, -0.15) | 1.11 (1.00, 1.22) | 0.044 |  | 0.94 | -0.47 (-0.85, -0.08) | 1.20 (1.01, 1.43) | 0.040 |
| Categorical SLEs |  |  |  |  |  |  |  |  |  |  |  |  |  |  |
| No SLEs | 2.52 | 0 (reference) | 1 (reference) |  |  | 2.11 | 0 (reference) | 1 (reference) |  |  | 0.65 | 0 (reference) | 1 (reference) |  |
| 1-2 SLEs | 3.18 | -1.36 (-2.15, -0.57) | 1.11 (1.01, 1.21) | 0.029 |  | 2.55 | -0.71 (-1.37, -0.06) | 1.11 (1.00, 1.22) | 0.053 |  | 0.95 | -0.44 (-0.84, -0.05) | 1.20 (1.01, 1.43) | 0.040 |
| SLEs > 2 | 3.51 | -3.92 (-7.15, -0.69) | 1.56 (1.10, 2.22) | 0.013 |  | 2.85 | -3.01 (-5.68, -0.34) | 1.20 (0.81, 1.77) | 0.362 |  | 0.88 | -1.07 (-2.67, 0.54) | 1.14 (0.56, 2.30) | 0.720 |
| Continuous SLEs |  |  |  |  |  |  |  |  |  |  |  |  |  |  |
| Per additional SLE | 2.77 | -0.95 (-1.49, -0.40) | 1.10 (1.04, 1.17) | 0.002 |  | 2.28 | -0.63 (-1.08, -0.17) | 1.09 (1.01, 1.16) | 0.018 |  | 0.76 | -0.22 (-0.49, 0.06) | 1.08 (0.96, 1.22) | 0.180 |
| **ELSA** |  |  |  |  |  |  |  |  |  |  |  |  |  |  |
| **Model 1** |  |  |  |  |  |  |  |  |  |  |  |  |  |  |
| Binary SLEs |  |  |  |  |  |  |  |  |  |  |  |  |  |  |
| No SLEs | 1.56 | 0 (reference) | 1 (reference) |  |  | 1.14 | 0 (reference) | 1 (reference) |  |  | 0.48 | 0 (reference) | 1 (reference) |  |
| SLEs | 2.45 | -0.37 (-0.58, -0.17) | 1.54 (1.32, 1.78) | < 0.001 |  | 1.81 | -0.19 (-0.28, -0.11) | 1.55 (1.30, 1.84) | < 0.001 |  | 0.81 | -0.10 (-0.17, -0.03) | 1.62 (1.24, 2.10) | < 0.001 |
| Categorical SLEs |  |  |  |  |  |  |  |  |  |  |  |  |  |  |
| No SLEs | 1.56 | 0 (reference) | 1 (reference) |  |  | 1.14 | 0 (reference) | 1 (reference) |  |  | 0.48 | 0 (reference) | 1 (reference) |  |
| 1-2 SLEs | 2.44 | -0.36 (-0.57, -0.16) | 1.53 (1.32, 1.78) | < 0.001 |  | 1.80 | -0.19 (-0.28, -0.10) | 1.54 (1.29, 1.83) | < 0.001 |  | 0.81 | -0.10 (-0.17, -0.03) | 1.63 (1.24, 2.12) | < 0.001 |
| SLEs > 2 | 2.93 | -0.64 (-1.36, 0.10) | 1.72 (1.07, 2.77) | 0.025 |  | 2.28 | -0.23 (-0.54, 0.08) | 1.81 (1.06, 3.11) | 0.031 |  | 0.81 | -0.08 (-0.33, 0.16) | 1.53 (0.62, 3.76) | 0.354 |
| Continuous SLEs |  |  |  |  |  |  |  |  |  |  |  |  |  |  |
| Per additional SLE | 1.98 | -0.22 (-0.36, -0.09) | 1.24 (1.13, 1.35) | < 0.001 |  | 1.46 | -0.10 (-0.16, -0.04) | 1.24 (1.12, 1.38) | < 0.001 |  | 0.64 | -0.05 (-0.10, -0.01) | 1.27 (1.08, 1.48) | 0.003 |
| **Model 2** |  |  |  |  |  |  |  |  |  |  |  |  |  |  |
| Binary SLEs |  |  |  |  |  |  |  |  |  |  |  |  |  |  |
| No SLEs | 1.56 | 0 (reference) | 1 (reference) |  |  | 1.14 | 0 (reference) | 1 (reference) |  |  | 0.48 | 0 (reference) | 1 (reference) |  |
| SLEs | 2.45 | -0.28 (-0.49, -0.08) | 1.38 (1.19, 1.61) | < 0.001 |  | 1.81 | -0.15 (-0.24, -0.07) | 1.42 (1.19, 1.69) | < 0.001 |  | 0.81 | -0.07 (-0.14, -0.01) | 1.39 (1.06, 1.83) | 0.016 |
| Categorical SLEs |  |  |  |  |  |  |  |  |  |  |  |  |  |  |
| No SLEs | 1.56 | 0 (reference) | 1 (reference) |  |  | 1.14 | 0 (reference) | 1 (reference) |  |  | 0.48 | 0 (reference) | 1 (reference) |  |
| 1-2 SLEs | 2.44 | -0.27 (-0.48, -0.06) | 1.37 (1.18, 1.60) | < 0.001 |  | 1.80 | -0.15 (-0.24, -0.06) | 1.41 (1.18, 1.68) | < 0.001 |  | 0.81 | -0.07 (-0.14, -0.01) | 1.40 (1.06, 1.83) | 0.017 |
| SLEs > 2 | 2.93 | -0.54 (-1.27, 0.19) | 1.55 (0.96, 2.51) | 0.071 |  | 2.28 | -0.20 (-0.51, 0.11) | 1.68 (0.98, 2.89) | 0.061 |  | 0.81 | -0.06 (-0.31, 0.19) | 1.34 (0.54, 3.31) | 0.526 |
| Continuous SLEs |  |  |  |  |  |  |  |  |  |  |  |  |  |  |
| Per additional SLE | 1.98 | -0.16 (-0.30, -0.03) | 1.15 (1.05, 1.27) | 0.003 |  | 1.46 | -0.07 (-0.13, -0.02) | 1.17 (1.05, 1.31) | 0.004 |  | 0.64 | -0.03 (-0.08, 0.01) | 1.15 (0.97, 1.36) | 0.100 |

Model 1 was unadjusted.

Model 2 was adjusted for age, sex, education, current drinking status, body mass index, hypertension, and diabetes.

CI, confidence interval; ELSA, English Longitudinal Study of Ageing; HR, hazard ratio; HRS, Health and Retirement Study; RMST, restricted mean survival time; SLEs, stressful life events.

**Supplementary Table S6. Association of adulthood stressful life events exposure with risks of incident cardiovascular diseases using data not being imputed**

| **All participants** | **Cardiovascular disease** | | | |  | **Heart disease** | | | |  | **Stroke** | | | |
| --- | --- | --- | --- | --- | --- | --- | --- | --- | --- | --- | --- | --- | --- | --- |
|  | **Event rate (events/100 person years)** | **RMST difference**  **(95% CI)** | **Cox models** | |  | **Event rate (events/100 person years)** | **RMST difference**  **(95% CI)** | **Cox models** | |  | **Event rate (events/100 person years)** | **RMST difference**  **(95% CI)** | **Cox models** | |
|  |  |  | **HR (95% CI )** | **P value** |  |  |  | **HR (95% CI )** | **P value** |  |  |  | **HR (95% CI )** | **P value** |
| **Model 2** |  |  |  |  |  |  |  |  |  |  |  |  |  |  |
| Binary SLEs |  |  |  |  |  |  |  |  |  |  |  |  |  |  |
| No SLEs | 2.37 | 0 (reference) | 1 (reference) |  |  | 1.96 | 0 (reference) | 1 (reference) |  |  | 0.62 | 0 (reference) | 1 (reference) |  |
| SLEs | 3.12 | -1.97 (-2.71, -1.23) | 1.18 (1.09, 1.28) | < 0.001 |  | 2.46 | -1.31 (-1.95, -0.68) | 1.14 (1.04, 1.25) | 0.006 |  | 0.95 | -0.70 (-1.11, -0.30) | 1.23 (1.06, 1.44) | 0.008 |
| Categorical SLEs |  |  |  |  |  |  |  |  |  |  |  |  |  |  |
| No SLEs | 2.37 | 0 (reference) | 1 (reference) |  |  | 1.96 | 0 (reference) | 1 (reference) |  |  | 0.62 | 0 (reference) | 1 (reference) |  |
| 1-2 SLEs | 3.11 | -1.87 (-2.61, -1.13) | 1.17 (1.08, 1.28) | < 0.001 |  | 2.45 | -1.24 (-1.87, -0.61) | 1.13 (1.03, 1.24) | 0.008 |  | 0.95 | -0.68 (-1.08, -0.28) | 1.23 (1.06, 1.44) | 0.008 |
| SLEs > 2 | 3.64 | -4.68 (-7.71, -1.66) | 1.43 (1.05, 1.95) | 0.025 |  | 2.84 | -3.40 (-5.96, -0.84) | 1.27 (0.89, 1.80) | 0.192 |  | 0.98 | -1.37 (-3.01, 0.27) | 1.29 (0.71, 2.35) | 0.408 |
| Continuous SLEs |  |  |  |  |  |  |  |  |  |  |  |  |  |  |
| Per additional SLE | 2.66 | -1.23 (-1.74, -0.71) | 1.11 (1.05, 1.17) | < 0.001 |  | 2.15 | -0.90 (-1.34, -0.46) | 1.09 (1.03, 1.16) | 0.005 |  | 0.75 | -0.35 (-0.63, -0.07) | 1.10 (0.99, 1.22) | 0.072 |

Model 2 was adjusted for age, sex, education, current drinking status, body mass index, hypertension, and diabetes.

CI, confidence interval; ELSA, English Longitudinal Study of Ageing; HR, hazard ratio; HRS, Health and Retirement Study; RMST, restricted mean survival time; SLEs, stressful life events.

**Supplementary Table S7. Association of adulthood stressful life events exposure with risks of incident cardiovascular diseases with additional adjustment for anti-hypertensive and anti-diabetic medications**

| **All participants** | **Cardiovascular disease** | | | |  | **Heart disease** | | | |  | **Stroke** | | | |
| --- | --- | --- | --- | --- | --- | --- | --- | --- | --- | --- | --- | --- | --- | --- |
|  | **Event rate (events/100 person years)** | **RMST difference**  **(95% CI)** | **Cox models** | |  | **Event rate (events/100 person years)** | **RMST difference**  **(95% CI)** | **Cox models** | |  | **Event rate (events/100 person years)** | **RMST difference**  **(95% CI)** | **Cox models** | |
|  |  |  | **HR (95% CI )** | **P value** |  |  |  | **HR (95% CI )** | **P value** |  |  |  | **HR (95% CI )** | **P value** |
| **Model 3** |  |  |  |  |  |  |  |  |  |  |  |  |  |  |
| Binary SLEs |  |  |  |  |  |  |  |  |  |  |  |  |  |  |
| No SLEs | 2.24 | 0 (reference) | 1 (reference) |  |  | 1.83 | 0 (reference) | 1 (reference) |  |  | 0.60 | 0 (reference) | 1 (reference) |  |
| SLEs | 2.91 | -2.22 (-2.91, -1.52) | 1.20 (1.11, 1.30) | < 0.001 |  | 2.27 | -1.57 (-2.17, -0.98) | 1.17 (1.07, 1.27) | < 0.001 |  | 0.89 | -0.79 (-1.17, -0.41) | 1.26 (1.09, 1.45) | 0.002 |
| Categorical SLEs |  |  |  |  |  |  |  |  |  |  |  |  |  |  |
| No SLEs | 2.24 | 0 (reference) | 1 (reference) |  |  | 1.83 | 0 (reference) | 1 (reference) |  |  | 0.60 | 0 (reference) | 1 (reference) |  |
| 1-2 SLEs | 2.90 | -2.13 (-2.83, -1.44) | 1.20 (1.11, 1.29) | < 0.001 |  | 2.26 | -1.50 (-2.09, -0.91) | 1.16 (1.06, 1.27) | < 0.001 |  | 0.90 | -0.77 (-1.15, -0.39) | 1.26 (1.09, 1.46) | 0.002 |
| SLEs > 2 | 3.27 | -4.42 (-7.13, -1.72) | 1.39 (1.05, 1.85) | 0.022 |  | 2.62 | -3.59 (-5.90, -1.29) | 1.33 (0.97, 1.82) | 0.079 |  | 0.85 | -1.17 (-2.66, 0.32) | 1.19 (0.68, 2.08) | 0.535 |
| Continuous SLEs |  |  |  |  |  |  |  |  |  |  |  |  |  |  |
| Per additional SLE | 2.51 | -1.34 (-1.81, -0.86) | 1.11 (1.06, 1.17) | < 0.001 |  | 2.01 | -1.01 (-1.42, -0.61) | 1.10 (1.04, 1.17) | < 0.001 |  | 0.72 | -0.42 (-0.68, -0.16) | 1.11 (1.01, 1.22) | 0.033 |

Model 3 was adjusted for age, sex, education, body mass index, hypertension, diabetes, anti-hypertensive and anti-diabetic medications.

CI, confidence interval; ELSA, English Longitudinal Study of Ageing; HR, hazard ratio; HRS, Health and Retirement Study; RMST, restricted mean survival time; SLEs, stressful life events.

**Supplementary Table S8. Association of adulthood stressful life events exposure with risks of incident cardiovascular diseases using the competing risk model**

| **All participants** | **Cardiovascular disease** | |  | **Heart disease** | |  | **Stroke** | |
| --- | --- | --- | --- | --- | --- | --- | --- | --- |
|  | **HR (95% CI )** | **P value** |  | **HR (95% CI )** | **P value** |  | **HR (95% CI )** | **P value** |
| **Model 2** |  |  |  |  |  |  |  |  |
| Binary SLEs |  |  |  |  |  |  |  |  |
| No SLEs | 1 (reference) |  |  | 1 (reference) |  |  | 1 (reference) |  |
| SLEs | 1.19 (1.10, 1.28) | < 0.001 |  | 1.09 (1.00, 1.20) | 0.062 |  | 1.21 (1.03, 1.42) | 0.022 |
| Categorical SLEs |  |  |  |  |  |  |  |  |
| No SLEs | 1 (reference) |  |  | 1 (reference) |  |  | 1 (reference) |  |
| 1-2 SLEs | 1.18 (1.09, 1.28) | < 0.001 |  | 1.09 (1.00, 1.20) | 0.063 |  | 1.21 (1.03, 1.43) | 0.020 |
| SLEs > 2 | 1.31 (0.98, 1.75) | 0.069 |  | 1.07 (0.74, 1.56) | 0.700 |  | 1.07 (0.56, 2.03) | 0.840 |
| Continuous SLEs |  |  |  |  |  |  |  |  |
| Per additional SLE | 1.10 (1.04, 1.16) | < 0.001 |  | 1.05 (0.99, 1.12) | 0.100 |  | 1.08 (0.97, 1.19) | 0.150 |

Model 2 was adjusted for age, sex, education, current drinking status, body mass index, hypertension, and diabetes.

CI, confidence interval; ELSA, English Longitudinal Study of Ageing; HR, hazard ratio; HRS, Health and Retirement Study; SLEs, stressful life events.

**Supplementary Table S9. Association of adulthood stressful life events exposure with risks of incident cardiovascular diseases at different follow-up time-points**

| **All participants** | **Cardiovascular disease** | | | |  | **Heart disease** | | | |  | **Stroke** | | | |
| --- | --- | --- | --- | --- | --- | --- | --- | --- | --- | --- | --- | --- | --- | --- |
|  | **Event rate (events/100 person years)** | **RMST difference**  **(95% CI)** | **Cox models** | |  | **Event rate (events/100 person years)** | **RMST difference**  **(95% CI)** | **Cox models** | |  | **Event rate (events/100 person years)** | **RMST difference**  **(95% CI)** | **Cox models** | |
|  |  |  | **HR (95% CI )** | **P value** |  |  |  | **HR (95% CI )** | **P value** |  |  |  | **HR (95% CI )** | **P value** |
| **Follow-up at the 3-year time point** | | | | | | | | | | | | | | |
| Binary SLEs |  |  |  |  |  |  |  |  |  |  |  |  |  |  |
| No SLEs | 3.53 | 0 (reference) | 1 (reference) |  |  | 2.87 | 0 (reference) | 1 (reference) |  |  | 0.92 | 0 (reference) | 1 (reference) |  |
| SLEs | 4.73 | -1.99 (-4.42, 0.44) | 1.24 (1.01, 1.54) | 0.043 |  | 3.53 | -0.97 (-3.10, 1.17) | 1.16 (0.91, 1.47) | 0.234 |  | 1.70 | -2.41 (-3.90, -0.92) | 1.66 (1.13, 2.44) | 0.010 |
| Categorical SLEs |  |  |  |  |  |  |  |  |  |  |  |  |  |  |
| No SLEs | 3.53 | 0 (reference) | 1 (reference) |  |  | 2.87 | 0 (reference) | 1 (reference) |  |  | 0.92 | 0 (reference) | 1 (reference) |  |
| 1-2 SLEs | 4.69 | -1.79 (-4.25, 0.67) | 1.23 (0.99, 1.52) | 0.057 |  | 3.44 | -0.60 (-2.75, 1.57) | 1.12 (0.88, 1.43) | 0.344 |  | 1.74 | -2.52 (-4.03, -1.01) | 1.69 (1.15, 2.48) | 0.008 |
| SLEs > 2 | 5.81 | -6.37 (-14.98, 2.23) | 1.66 (0.82, 3.40) | 0.162 |  | 5.81 | -9.05 (-16.61, -1.49) | 2.13 (1.04, 4.36) | 0.039 |  | 0.73 | -0.09 (-5.38, 5.19) | 0.79 (0.11, 5.74) | 0.812 |
| Continuous SLEs |  |  |  |  |  |  |  |  |  |  |  |  |  |  |
| Per additional SLE | 4.03 | -0.99 (-2.61, 0.64) | 1.10 (0.96, 1.27) |  |  | 3.15 | -0.80 (-2.23, 0.63) | 1.10 (0.94, 1.28) | 0.250 |  | 1.25 | -0.96 (-1.96, 0.04) | 1.18 (0.92, 1.51) | 0.185 |
| **Follow-up at the 6-year time point** | | | | | | | | | | | | | | |
| Binary SLEs |  |  |  |  |  |  |  |  |  |  |  |  |  |  |
| No SLEs | 2.90 | 0 (reference) | 1 (reference) |  |  | 2.27 | 0 (reference) | 1 (reference) |  |  | 0.89 | 0 (reference) | 1 (reference) |  |
| SLEs | 4.05 | -2.52 (-4.16, -0.88) | 1.29 (1.10, 1.51) | 0.002 |  | 3.16 | -1.98 (-3.35, -0.62) | 1.29 (1.08, 1.54) | 0.005 |  | 1.28 | -0.89 (-1.82, 0.05) | 1.24 (0.94, 1.65) | 0.136 |
| Categorical SLEs |  |  |  |  |  |  |  |  |  |  |  |  |  |  |
| No SLEs | 2.90 | 0 (reference) | 1 (reference) |  |  | 2.27 | 0 (reference) | 1 (reference) |  |  | 0.89 | 0 (reference) | 1 (reference) |  |
| 1-2 SLEs | 4.06 | -2.49 (-4.15, -0.83) | 1.29 (1.10, 1.51) | 0.001 |  | 3.14 | -1.87 (-3.25, -0.49) | 1.29 (1.08, 1.54) | 0.006 |  | 1.31 | -0.97 (-1.91, -0.03) | 1.27 (0.95, 1.68) | 0.102 |
| SLEs > 2 | 3.92 | -3.23 (-9.10, 2.63) | 1.18 (0.68, 2.06) | 0.560 |  | 3.62 | -4.47 (-9.35, 0.41) | 1.37 (0.77, 2.45) | 0.288 |  | 0.60 | 1.01 (-2.32, 4.35) | 0.58 (0.14, 2.35) | 0.442 |
| Continuous SLEs |  |  |  |  |  |  |  |  |  |  |  |  |  |  |
| Per additional SLE | 3.40 | -1.49 (-2.60, -0.38) | 1.14 (1.03, 1.26) | 0.012 |  | 2.66 | -1.38 (-2.31, -0.46) | 1.16 (1.04, 1.30) | 0.008 |  | 1.06 | -0.33 (-0.97, 0.30) | 1.06 (0.88, 1,27) | 0.574 |
| **Follow-up at the 9-year time point** | | | | | | | | | | | | | | |
| Binary SLEs |  |  |  |  |  |  |  |  |  |  |  |  |  |  |
| No SLEs | 2,56 | 0 (reference) | 1 (reference) |  |  | 2.12 | 0 (reference) | 1 (reference) |  |  | 0.67 | 0 (reference) | 1 (reference) |  |
| SLEs | 3.33 | -2.00 (-2.78, -1.22) | 1.19 (1.10, 1.30) | < 0.001 |  | 2.63 | -1.28 (-1.94, -0.62) | 1.15 (1.04, 1.26) | 0.004 |  | 0.99 | -0.67 (-1.08, -0.27) | 1.26 (1.07, 1.47) | 0.005 |
| Categorical SLEs |  |  |  |  |  |  |  |  |  |  |  |  |  |  |
| No SLEs | 2.56 | 0 (reference) | 1 (reference) |  |  | 2.12 | 0 (reference) | 1 (reference) |  |  | 0.67 | 0 (reference) | 1 (reference) |  |
| 1-2 SLEs | 3.32 | -1.92 (-2.69, -1.14) | 1.19 (1.09, 12.9） | < 0.001 |  | 2.62 | -1.20 (-1.85, -0.54) | 1.14 (1.04, 1.25) | 0.006 |  | 1.00 | -0.70 (-1.07, -0.27) | 1.26 (1.07, 1.48) | 0.005 |
| SLEs > 2 | 3.60 | -4.05 (-7.09, -1.00) | 1.39 (1.01, 1.91) | 0.045 |  | 2.96 | -3.44 (-6.02, -0.87) | 1.28 (0.90, 1.81) | 0.177 |  | 0.83 | -0.75 (-2.34, -0.83) | 1.09 (0.56, 2.11) | 0.803 |
| Continuous SLEs |  |  |  |  |  |  |  |  |  |  |  |  |  |  |
| Per additional SLE | 2.85 | -1.28 (-1.82, -0.75) | 1.12 (1.06, 1.18) | < 0.001 |  | 2.31 | -0.95 (-1.40, -0.50) | 1.11 (1.04, 1.18) | 0.001 |  | 0.79 | -0.31 (-0.58, -0.03) | 1.11 (0.99, 1.23) | 0.068 |

All models were adjusted for age, sex, education, current drinking status, body mass index, hypertension, and diabetes.

CI, confidence interval; ELSA, English Longitudinal Study of Ageing; HR, hazard ratio; HRS, Health and Retirement Study; RMST, restricted mean survival time; SLEs, stressful life events.

**Supplementary Table S10. Association of adulthood stressful life events exposure with risks of incident cardiovascular diseases excluding participants who were diagnosed with CVD within the first 2 years of follow-up**

| **All participants** | **Cardiovascular disease** | | | |  | **Heart disease** | | | |  | **Stroke** | | | |
| --- | --- | --- | --- | --- | --- | --- | --- | --- | --- | --- | --- | --- | --- | --- |
|  | **Event rate (events/100 person years)** | **RMST difference**  **(95% CI)** | **Cox models** | |  | **Event rate (events/100 person years)** | **RMST difference**  **(95% CI)** | **Cox models** | |  | **Event rate (events/100 person years)** | **RMST difference**  **(95% CI)** | **Cox models** | |
|  |  |  | **HR (95% CI )** | **P value** |  |  |  | **HR (95% CI )** | **P value** |  |  |  | **HR (95% CI )** | **P value** |
| **Model 2** |  |  |  |  |  |  |  |  |  |  |  |  |  |  |
| Binary SLEs |  |  |  |  |  |  |  |  |  |  |  |  |  |  |
| No SLEs | 1.64 | 0 (reference) | 1 (reference) |  |  | 1.32 | 0 (reference) | 1 (reference) |  |  | 0.44 | 0 (reference) | 1 (reference) |  |
| SLEs | 2.01 | -1.31 (-1.84, -0.78) | 1.15 (1.05, 1.26) | 0.003 |  | 1.57 | -1.00 (-1.47, -0.52) | 1.13 (1.02, 1.25) | 0.021 |  | 0.62 | -0.48 (-0.78, -0.17) | 1.21 (1.02, 1.44) | 0.030 |
| Categorical SLEs |  |  |  |  |  |  |  |  |  |  |  |  |  |  |
| No SLEs | 1.64 | 0 (reference) | 1 (reference) |  |  | 1.32 | 0 (reference) | 1 (reference) |  |  | 0.44 | 0 (reference) | 1 (reference) |  |
| 1-2 SLEs | 1.99 | -1.22 (-1.76, -0.69) | 1.14 (1.04, 1.26) | 0.005 |  | 1.56 | -0.95 (-1.43, -0.48) | 1.13 (1.02, 1.25) | 0.025 |  | 0.61 | -0.42 (-0.73, -0.12) | 1.20 (1.00, 1.43) | 0.044 |
| SLEs > 2 | 2.37 | -3.79 (-5.92, -1.67) | 1.43 (1.02, 2.01) | 0.037 |  | 1.69 | -2.09 (-3.99, -0.19) | 1.20 (0.81, 1.79) | 0.362 |  | 0.81 | -1.90 (-3.11, -0.69) | 1.63 (0.91, 2.92) | 0.098 |
| Continuous SLEs |  |  |  |  |  |  |  |  |  |  |  |  |  |  |
| Per additional SLE | 1.78 | -0.86 (-1.23, -0.49) | 1.09 (1.03, 1.16) | 0.005 |  | 1.42 | -0.62 (-0.95, -0.29) | 1.08 (1.01, 1.16) | 0.028 |  | 0.51 | -0.32 (-0.53, -0.11) | 1.12 (1.00, 1.26) | 0.052 |

Model 2 was adjusted for age, sex, education, current drinking status, body mass index, hypertension, and diabetes.

CI, confidence interval; ELSA, English Longitudinal Study of Ageing; HR, hazard ratio; HRS, Health and Retirement Study; RMST, restricted mean survival time; SLEs, stressful life events.

**Supplementary Table S11. Association of adulthood stressful life events exposure with risks of incident cardiovascular diseases excluding participants with low related to self-report accuracy**

| **All participants** | **Cardiovascular disease** | | | |  | **Heart disease** | | | |  | **Stroke** | | | |
| --- | --- | --- | --- | --- | --- | --- | --- | --- | --- | --- | --- | --- | --- | --- |
|  | **Event rate (events/100 person years)** | **RMST difference**  **(95% CI)** | **Cox models** | |  | **Event rate (events/100 person years)** | **RMST difference**  **(95% CI)** | **Cox models** | |  | **Event rate (events/100 person years)** | **RMST difference**  **(95% CI)** | **Cox models** | |
|  |  |  | **HR (95% CI )** | **P value** |  |  |  | **HR (95% CI )** | **P value** |  |  |  | **HR (95% CI )** | **P value** |
| **Excluding participants aged ≥75 years** | | | | | | | | | | | | | | |
| Binary SLEs |  |  |  |  |  |  |  |  |  |  |  |  |  |  |
| No SLEs | 1.98 | 0 (reference) | 1 (reference) |  |  | 1.61 | 0 (reference) | 1 (reference) |  |  | 0.49 | 0 (reference) | 1 (reference) |  |
| SLEs | 2.51 | -2.41 (-3.15, -1.67) | 1.27 (1.16, 1.39) | < 0.001 |  | 2.02 | -1.83 (-2.45, -1.20) | 1.26 (1.14, 1.39) | < 0.001 |  | 0.69 | -0.76 (-1.14, -0.38) | 1.34 (1.12, 1.60) | 0.001 |
| Categorical SLEs |  |  |  |  |  |  |  |  |  |  |  |  |  |  |
| No SLEs | 1.98 | 0 (reference) | 1 (reference) |  |  | 1.61 | 0 (reference) | 1 (reference) |  |  | 0.49 | 0 (reference) | 1 (reference) |  |
| 1-2 SLEs | 2.49 | -2.32 (-3.07, -1.57) | 1.26 (1.15, 1.38) | < 0.001 |  | 2.00 | -1.74 (-2.38, -1.11) | 1.25 (1.13, 1.39) | < 0.001 |  | 0.69 | -0.74 (-1.13, -0.36) | 1.34 (1.12, 1.60) | 0.001 |
| SLEs > 2 | 2.98 | -4.70 (-7.66, -1.74) | 1.52 (1.09, 2.12) | 0.014 |  | 2.40 | -3.93 (-6.44, -1.42) | 1.46 (1.01, 2.12) | 0.045 |  | 0.74 | -1.14 (-2.66, 0.38) | 1.43 (0.74, 2.79) | 0.290 |
| Continuous SLEs |  |  |  |  |  |  |  |  |  |  |  |  |  |  |
| Per additional SLE | 2.17 | -1.52 (-2.03, -1.00) | 1.15 (1.09, 1.23) | < 0.001 |  | 1.76 | -1.18 (-1.62, -0.74) | 1.16 (1.08, 1.24) | < 0.001 |  | 0.57 | -0.43 (-0.69, -0.16) | 1.17 (1.04, 1.31) | 0.008 |
| **Excluding participants with education below high school** | | | | | | | | | | | | | | |
| Binary SLEs |  |  |  |  |  |  |  |  |  |  |  |  |  |  |
| No SLEs | 2.23 | 0 (reference) | 1 (reference) |  |  | 1.86 | 0 (reference) | 1 (reference) |  |  | 0.55 | 0 (reference) | 1 (reference) |  |
| SLEs | 2.97 | -2.38 (-3.14, -1.62) | 1.23 (1.13, 1.34) | < 0.001 |  | 2.33 | -1.58 (-2.24, -0.92) | 1.16 (1.06, 1.28) | 0.002 |  | 0.86 | -0.89 (-1.29, -0.49) | 1.32 (1.11, 1.56) | 0.001 |
| Categorical SLEs |  |  |  |  |  |  |  |  |  |  |  |  |  |  |
| No SLEs | 2.23 | 0 (reference) | 1 (reference) |  |  | 1.86 | 0 (reference) | 1 (reference) |  |  | 0.55 | 0 (reference) | 1 (reference) |  |
| 1-2 SLEs | 2.96 | -2.35 (-3.12, -1.58) | 1.23 (1.12, 1.34) | < 0.001 |  | 2.32 | -1.54 (-2.20, -0.87) | 1.16 (1.05, 1.28) | 0.003 |  | 0.87 | -0.89 (-1.30, -0.49) | 1.32 (1.12, 1.57) | 0.001 |
| SLEs > 2 | 3.13 | -3.32 (-6.60, -0.05) | 1.38 (0.97, 1.97) | 0.077 |  | 2.62 | -2.84 (-5.68, -0.01) | 1.31 (0.89, 1.94) | 0.169 |  | 0.71 | -0.82 (-2.54, 0.90) | 1.13 (0.53, 2.39) | 0.753 |
| Continuous SLEs |  |  |  |  |  |  |  |  |  |  |  |  |  |  |
| Per additional SLE | 2.50 | -1.45 (-1.99, -0.91) | 1.14 (1.07, 1.21) | < 0.001 |  | 2.03 | -1.03 (-1.50, -0.56) | 1.12 (1.05, 1.20) | < 0.001 |  | 0.67 | -0.50 (-0.78, -0.21) | 1.16 (1.04, 1.30) | 0.011 |
| **Excluding participants cognitive score in the lowest quartile** | | | | | | | | | | | | | | |
| Binary SLEs |  |  |  |  |  |  |  |  |  |  |  |  |  |  |
| No SLEs | 2.07 | 0 (reference) | 1 (reference) |  |  | 1.73 | 0 (reference) | 1 (reference) |  |  | 0.49 | 0 (reference) | 1 (reference) |  |
| SLEs | 2.89 | -3.11 (-3.90, -2.33) | 1.32 (1.20, 1.45) | < 0.001 |  | 2.33 | -2.19 (-2.87, -1.51) | 1.27 (1.14, 1.40) | < 0.001 |  | 0.77 | -1.05 (-1.45, -0.65) | 1.39 (1.16, 1.67) | < 0.001 |
| Categorical SLEs |  |  |  |  |  |  |  |  |  |  |  |  |  |  |
| No SLEs | 2.07 | 0 (reference) | 1 (reference) |  |  | 1.73 | 0 (reference) | 1 (reference) |  |  | 0.49 | 0 (reference) | 1 (reference) |  |
| 1-2 SLEs | 2.87 | -3.01 (-3.80, -2.22) | 1.31 (1.19, 1.43) | < 0.001 |  | 2.31 | -2.09 (-2.78, -1.41) | 1.25 (1.13, 1.39) | < 0.001 |  | 0.77 | -1.03 (-1.44, -0.62) | 1.39 (1.16, 1.67) | < 0.001 |
| SLEs > 2 | 3.50 | -6.06 (-9.39, -2.73) | 1.67 (1.18, 2.37) | 0.004 |  | 2.97 | -4.77 (-7.67, -1.88) | 1.65 (1.13, 2.40) | 0.010 |  | 0.74 | -1.61 (-3.31, 0.10) | 1.40 (0.66, 2.97) | 0.379 |
| Continuous SLEs |  |  |  |  |  |  |  |  |  |  |  |  |  |  |
| Per additional SLE | 2.37 | -1.96 (-2.52, -1.41) | 1.19 (1.12, 1.26) | < 0.001 |  | 1.95 | -1.47 (-1.95, -0.98) | 1.18 (1.10, 1.26) | < 0.001 |  | 0.59 | -0.59 (-0.88, -0.31) | 1.17 (1.04, 1.33) | 0.012 |

All models were adjusted for age, sex, education, current drinking status, body mass index, hypertension, and diabetes.

CI, confidence interval; ELSA, English Longitudinal Study of Ageing; HR, hazard ratio; HRS, Health and Retirement Study; RMST, restricted mean survival time; SLEs, stressful life events.

**Supplementary Table S12. Mediation analysis employing second-wave measurements**

|  | **Cardiovascular disease** | |  | **Heart disease** | |  | **Stroke** | |
| --- | --- | --- | --- | --- | --- | --- | --- | --- |
|  | **RMST difference**  **(95% CI), months** | **PERM, %** |  | **RMST difference**  **(95% CI)** | **PERM, %** |  | **RMST difference**  **(95% CI)** | **PERM, %** |
| **Depressive symptoms^¶^** |  |  |  |  |  |  |  |  |
| No-exposure | 0 (reference) |  |  | 0 (reference) |  |  | 0 (reference) |  |
| Exposure, multivariate model | -1.84 (-2.50, -1.18) |  |  | -1.19 (-1.71, -0.68) |  |  | -0.61 (-0.95, -0.28) |  |
| Exposure, depressive symptoms adjusted | -1.73 (-2.40, -1.07) | 5.98 |  | -1.15 (-1.67, -0.63) | 3.36 |  | -0.57 (-0.91, -0.24) | 6.56 |
| **Physical activity^¶^** |  |  |  |  |  |  |  |  |
| No-exposure | 0 (reference) |  |  | 0 (reference) |  |  | 0 (reference) |  |
| Exposure, multivariate model | -1.84 (-2.50, -1.18) |  |  | -1.19 (-1.71, -0.68) |  |  | -0.61 (-0.95, -0.28) |  |
| Exposure, physical activity adjusted | -1.78 (-2.44, -1.12) | 3.26 |  | -1.16 (-1.67, -0.64) | 2.52 |  | -0.57 (-0.91, -0.24) | 6.56 |
| **Current smoking^¶^** |  |  |  |  |  |  |  |  |
| No-exposure | 0 (reference) |  |  | 0 (reference) |  |  | 0 (reference) |  |
| Exposure, multivariate model | -1.84 (-2.50, -1.18) |  |  | -1.19 (-1.71, -0.68) |  |  | -0.61 (-0.95, -0.28) |  |
| Exposure, current smoking adjusted | -1.81 (-2.47, -1.15) | 1.63 |  | -1.18 (-1.70, -0.67) | 0.84 |  | -0.60 (-0.94, -0.26) | 1.64 |

**^¶^** Multivariate model was adjusted for age, sex, education,drinking status, body mass index, hypertension, and diabetes.

CI, confidence interval; HR, hazard ratio; PERM, percentage of excess risk mediated; SLEs, stressful life events; RMST, restricted mean survival time.

**Supplementary Table S13. Association of each adulthood stressful life event component with incident cardiovascular outcomes**

| **All participants** | **Cardiovascular disease** | | | |  | **Heart disease** | | |  | **Stroke** | | |
| --- | --- | --- | --- | --- | --- | --- | --- | --- | --- | --- | --- | --- |
|  | **RMST difference**  **(95% CI)** | **Cox models** | | |  | **RMST difference**  **(95% CI)** | **Cox models** | |  | **RMST difference**  **(95% CI)** | **Cox models** | |
|  |  | **HR (95% CI )** | **P value** | |  |  | **HR (95% CI )** | **P value** |  |  | **HR (95% CI )** | **P value** |
| **Model 1** |  |  | |  |  |  |  |  |  |  |  |  |
| Unemployment vs. employment | 1.79 (-0.15, 3.73) | 0.89 (0.71, 1.13) | | 0.340 |  | 1.75 (0.10, 3.40) | 0.89 (0.69, 1.14) | 0.355 |  | 2.45 (0.12, 4.78) | 0.70 (0.43, 1.13) | 0.701 |
| Asset poverty vs. asset sufficiency | 0.18 (-0.81, 1.18) | 0.94 (0.83, 1.05) | | 0.248 |  | -0.07 (-0.91, 0.78) | 0.95 (0.83, 1.07) | 0.378 |  | 0.84 (-0.36, 2.03) | 0.92 (0.74, 1.14) | 0.438 |
| Death of a child vs. no child death | -3.14 (-4.63, -1.64) | 1.34 (1.15, 1.56) | | < 0.001 |  | -2.35 (-3.62, -1.08) | 1.36 (1.15, 1.61) | < 0.001 |  | -1.33 (-3.13, 0.47) | 1.48 (1.13, 1.94) | 0.004 |
| Death of a spouse/partner vs. no spouse/partner death | -3.08 (-4.02, -2.15) | 1.42 (1.29, 1.56) | | < 0.001 |  | -1.76 (-2.56, -0.97) | 1.28 (1.15, 1.42) | < 0.001 |  | -3.09 (-4.21, -1.97) | 1.76 (1.49, 2.08) | < 0.001 |
| Life-threatening illness/accident vs. no life-threatening illness/accident | -3.37 (-4.39, -2.35) | 1.21 (1.09, 1.35) | | < 0.001 |  | -2.63 (-3.50, -1.76) | 1.21 (1.07, 1.36) | 0.002 |  | -1.25 (-2.47, -0.02) | 1.28 (1.05, 1.56) | 0.013 |
| Physical attack/injury vs. no physical attack/injury | -2.54 (-4.32, -0.76) | 1.21 (1.01, 1.45) | | 0.042 |  | -2.62 (-4.14, -1.11) | 1.31 (1.08, 1.60) | 0.007 |  | 0.27 (-1.87, 2.41) | 0.90 (0.61, 1.34) | 0.612 |
| **Model 2** |  |  | |  |  |  |  |  |  |  |  |  |
| Unemployment vs. employment | -0.49 (-2.43, 1.45) | 1.19 (0.94, 1.50) | | 0.149 |  | 0.26 (-1.39, 1.92) | 1.14 (0.88, 1.48) | 0.315 |  | 0.89 (-1.46, 3.23) | 1.07 (0.66, 1.74) | 0.791 |
| Asset poverty vs. asset sufficiency | -0.84 (-1.86, 0.17) | 1.07 (0.96, 1.21) | | 0.231 |  | -0.72 (-1.58, 0.15) | 1.07 (0.94, 1.22) | 0.306 |  | 0.10 (-1.12, 1.33) | 1.06 (0.85, 1.33) | 0.582 |
| Death of a child vs. no child death | -1.64 (-3.13, -0.15) | 1.14 (0.97, 1.32) | | 0.104 |  | -1.36 (-2.64, -0.09) | 1.17 (0.99, 1.39) | 0.062 |  | -0.28 (-2.09, 1.52) | 1.17 (0.89, 1.53) | 0.271 |
| Death of a spouse/partner vs. no spouse/partner death | 0.06 (-0.97, 1.10) | 0.98 (0.88, 1.09) | | 0.737 |  | 0.24 (-0.64, 1.12) | 0.93 (0.83, 1.06) | 0.271 |  | -0.70 (-1.95, 0.56) | 1.02 (0.84, 1.23) | 0.844 |
| Life-threatening illness/accident vs. no life-threatening illness/accident | -3.17 (-4.18, -2.15) | 1.23 (1.10, 1.37) | | < 0.001 |  | -2.45 (-3.32, -1.59) | 1.22 (1.08, 1.38) | 0.001 |  | -1.29 (-2.52, -0.06) | 1.30 (1.07, 1.58) | 0.009 |
| Physical attack/injury vs. no physical attack/injury | -3.92 (-5.69, -2.15) | 1.44 (1.20, 1.74) | | < 0.001 |  | -3.51 (-5.02, -2.00) | 1.53 (1.25, 1.86) | < 0.001 |  | -0.81 (-2.95, 1.33) | 1.18 (0.80, 1.74) | 0.416 |

Model 1 was unadjusted.

Model 2 was adjusted for age, sex, education, body mass index, hypertension, and diabetes.

CI, confidence interval; ELSA, English Longitudinal Study of Ageing; HR, hazard ratio; HRS, Health and Retirement Study; RMST, restricted mean survival time.

**Supplementary Table S14. Association of depression-weighted stressful life event score with incident cardiovascular outcomes**

| **All participants** | **Cardiovascular disease** | | |  | **Heart disease** | | |  | **Stroke** | | |
| --- | --- | --- | --- | --- | --- | --- | --- | --- | --- | --- | --- |
|  | **RMST difference**  **(95% CI)** | **Cox models** | |  | **RMST difference**  **(95% CI)** | **Cox models** | |  | **RMST difference**  **(95% CI)** | **Cox models** | |
|  |  | **HR (95% CI )** | **P value** |  |  | **HR (95% CI )** | **P value** |  |  | **HR (95% CI )** | **P value** |
| **Model 1** |  |  |  |  |  |  |  |  |  |  |  |
| **Continuous analysis** |  |  |  |  |  |  |  |  |  |  |  |
| Per 1-score increase | -0.17 (-0.24, -0.09) | 1.01 (1.01, 1.02) | < 0.001 |  | -0.11 (-0.17, -0.06) | 1.01 (1.00, 1.02) | 0.009 |  | -0.05 (-0.09, -0.01) | 1.02 (1.01, 1.03) | 0.007 |
| Per 1-traumatic equivalent | -1.00 (-1.44, -0.56) | 1.09 (1.04, 1.14) | < 0.001 |  | -0.73 (-1.11, -0.36) | 1.07 (1.02, 1.13) | 0.009 |  | -0.32 (-0.56, -0.08) | 1.13 (1.03, 1.23) | 0.007 |
| **Categorical analysis** |  |  |  |  |  |  |  |  |  |  |  |
| No events | 1 (reference) | 1 (reference) |  |  | 1 (reference) | 1 (reference) |  |  | 1 (reference) | 1 (reference) |  |
| Low burden | -3.37 (-4.41, -2.32) | 1.27 (1.14, 1.42) | < 0.001 |  | -2.36 (-3.25, -1.47) | 1.22 (1.07, 1.38) | 0.002 |  | -1.48 (-2.05, -0.91) | 1.44 (1.17, 1.76) | < 0.001 |
| Moderate-high burden | -2.80 (-3.56, -2.05) | 1.31 (1.21, 1.43) | < 0.001 |  | -1.92 (-2.56, -1.28) | 1.25 (1.14, 1.37) | < 0.001 |  | -1.04 (-1.46, -0.63) | 1.50 (1.28, 1.74) | < 0.001 |
| **Model 2** |  |  |  |  |  |  |  |  |  |  |  |
| **Continuous analysis** |  |  |  |  |  |  |  |  |  |  |  |
| Per 1-score increase | -0.14 (-0.21, -0.06) | 1.01 (1.00, 1.02) | 0.006 |  | -0.10 (-0.16, -0.04) | 1.01 (1.00, 1.02) | 0.032 |  | -0.03 (-0.07, 0.01) | 1.01 (1.00, 1.02) | 0.208 |
| Per 1-traumatic equivalent | -0.82 (-1.27, -0.37) | 1.07 (1.02, 1.13) | 0.006 |  | -0.63 (-1.01, -0.24) | 1.06 (1.01, 1.12) | 0.032 |  | -0.19 (-0.44, 0.05) | 1.06 (0.97, 1.17) | 0.208 |
| **Categorical analysis** |  |  |  |  |  |  |  |  |  |  |  |
| No events | 1 (reference) | 1 (reference) |  |  | 1 (reference) | 1 (reference) |  |  | 1 (reference) | 1 (reference) |  |
| Low burden | -3.11 (-4.14, -2.07) | 1.27 (1.14, 1.42) | < 0.001 |  | -2.16 (-3.04, -1.28) | 1.22 (1.07, 1.38) | 0.002 |  | -1.35 (-1.91, -0.78) | 1.42 (1.16, 1.75) | < 0.001 |
| Moderate-high burden | -1.80 (-2.57, -1.02) | 1.17 (1.07, 1.28) | < 0.001 |  | -1.30 (-1.96, -0.64) | 1.14 (1.03, 1.26) | 0.008 |  | -0.53 (-0.95, -0.10) | 1.19 (1.01, 1.40) | 0.035 |

Model 1 was unadjusted.

Model 2 was adjusted for age, sex, education, body mass index, hypertension, and diabetes.

CI, confidence interval; ELSA, English Longitudinal Study of Ageing; HR, hazard ratio; HRS, Health and Retirement Study; RMST, restricted mean survival time.

**Supplementary Table S15. Association of adulthood stressful life events exposure with risks of incident cardiovascular diseases stratified by age**

| **All participants** | **Cardiovascular disease** | | | | |  | **Heart disease** | | | | |  | **Stroke** | | | | |
| --- | --- | --- | --- | --- | --- | --- | --- | --- | --- | --- | --- | --- | --- | --- | --- | --- | --- |
|  | **Event rate (events/100 person years)** | **RMST difference**  **(95% CI)** | **Cox models** | | |  | **Event rate (events/100 person years)** | **RMST difference**  **(95% CI)** | **Cox models** | | |  | **Event rate (events/100 person years)** | **RMST difference**  **(95% CI)** | **Cox models** | | |
|  |  |  | **HR (95% CI )** | **P value** | **P for interaction** |  |  |  | **HR (95% CI )** | **P value** | **P for interaction** |  |  |  | **HR (95% CI )** | **P value** | **P for interaction** |
| **Age <65 years** |  |  |  |  |  |  |  |  |  |  |  |  |  |  |  |  |  |
| Binary SLEs |  |  |  |  | 0.086 |  |  |  |  |  | 0.069 |  |  |  |  |  | 0.121 |
| No SLEs | 1.56 | 0 (reference) | 1 (reference) |  |  |  | 1.29 | 0 (reference) | 1 (reference) |  |  |  | 0.36 | 0 (reference) | 1 (reference) |  |  |
| SLEs | 2.07 | -2.23 (-3.06, -1.41) | 1.30 (1.16, 1.47) | < 0.001 |  |  | 1.69 | -1.62 (-2.32, -0.92) | 1.28 (1.13, 1.47) | < 0.001 |  |  | 0.54 | -0.67 (-1.06, -0.28) | 1.43 (1.12, 1.83) | 0.004 |  |
| Categorical SLEs |  |  |  |  | 0.226 |  |  |  |  |  | 0.193 |  |  |  |  |  | 0.274 |
| No SLEs | 1.56 | 0 (reference) | 1 (reference) |  |  |  | 1.29 | 0 (reference) | 1 (reference) |  |  |  | 0.36 | 0 (reference) | 1 (reference) |  |  |
| 1-2 SLEs | 2.06 | -2.15 (-2.97, -1.33) | 1.29 (1.15, 1.46) | < 0.001 |  |  | 1.68 | -1.55 (-2.24, -0.85) | 1.28 (1.12, 1.46) | < 0.001 |  |  | 0.45 | -0.67 (-1.07, -0.28) | 1.44 (1.13, 1.84) | 0.003 |  |
| SLEs > 2 | 2.45 | -4.14 (-7.26, -1.01) | 1.56 (1.02, 2.40) | 0.039 |  |  | 2.01 | -3.29 (-5.92, -0.63) | 1.49 (0.93, 2.39) | 0.096 |  |  | 0.55 | -0.55 (-2.05, 0.94) | 1.16 (0.43, 3.14) | 0.769 |  |
| Continuous SLEs |  |  |  |  | 0.079 |  |  |  |  |  | 0.127 |  |  |  |  |  | 0.197 |
| Per additional SLE | 1.75 | -1.33 (-1.89, -0.77) | 1.17 (1.08, 1.27) | < 0.001 |  |  | 1.44 | -0.93 (-1.41, -0.46) | 1.16 (1.07, 1.27) | < 0.001 |  |  | 0.43 | -0.32 (-0.59, -0.05) | 1.19 (1.02, 1.39) | 0.031 |  |
| **Age ≥75 years** |  |  |  |  |  |  |  |  |  |  |  |  |  |  |  |  |  |
| Binary SLEs |  |  |  |  | 0.086 |  |  |  |  |  | 0.069 |  |  |  |  |  | 0.121 |
| No SLEs | 3.29 | 0 (reference) | 1 (reference) |  |  |  | 2.66 | 0 (reference) | 1 (reference) |  |  |  | 0.97 | 0 (reference) | 1 (reference) |  |  |
| SLEs | 3.85 | -2.50 (-3.70, -1.29) | 1.17 (1.06, 1.30) | 0.002 |  |  | 2.93 | -1.75 (-2.78, -0.73) | 1.13 (1.00, 1.26) | 0.043 |  |  | 1.28 | -0.90 (-1.61, -0.19) | 1.20 (1.00, 1.44) | 0.051 |  |
| Categorical SLEs |  |  |  |  | 0.226 |  |  |  |  |  | 0.193 |  |  |  |  |  | 0.274 |
| No SLEs | 3.29 | 0 (reference) | 1 (reference) |  |  |  | 2.66 | 0 (reference) | 1 (reference) |  |  |  | 0.97 | 0 (reference) | 1 (reference) |  |  |
| 1-2 SLEs | 3.83 | -2.42 (-3.62, -1.23) | 1.17 (1.06, 1.29) | 0.003 |  |  | 2.91 | -1.68 (-2.70, -0.66) | 1.12 (1.00, 1.26) | 0.051 |  |  | 1.28 | -0.86 (-1.57, -0.16) | 1.20 (1.00, 1.43) | 0.055 |  |
| SLEs > 2 | 4.44 | -4.85 (-9.60, -0.10) | 1.31 (0.90, 1.91) | 0.164 |  |  | 3.49 | -4.11 (-8.14, -0.07) | 1.23 (0.80, 1.89) | 0.341 |  |  | 1.43 | -2.00 (-4.79, 0.80) | 1.27 (0.65, 2.47) | 0.490 |  |
| Continuous SLEs |  |  |  |  | 0.079 |  |  |  |  |  | 0.127 |  |  |  |  |  | 0.197 |
| Per additional SLE | 3.54 | -1.47 (-2.29, -0.65) | 1.09 (1.02, 1.17) | 0.013 |  |  | 2.78 | -1.24 (-1.93, -0.54) | 1.08 (1.00, 1.17) | 0.042 |  |  | 1.11 | -0.51 (-0.99, -0.03) | 1.09 (0.97, 1.23) | 0.167 |  |

Models were adjusted for age, sex, education, body mass index, hypertension, and diabetes.

CI, confidence interval; ELSA, English Longitudinal Study of Ageing; HR, hazard ratio; HRS, Health and Retirement Study; RMST, restricted mean survival time.

**Supplementary Table S16. Association of adulthood stressful life events exposure with risks of incident cardiovascular diseases stratified by sex**

| **All participants** | **Cardiovascular disease** | | | | |  | **Heart disease** | | | | |  | **Stroke** | | | | |
| --- | --- | --- | --- | --- | --- | --- | --- | --- | --- | --- | --- | --- | --- | --- | --- | --- | --- |
|  | **Event rate (events/100 person years)** | **RMST difference**  **(95% CI)** | **Cox models** | | |  | **Event rate (events/100 person years)** | **RMST difference**  **(95% CI)** | **Cox models** | | |  | **Event rate (events/100 person years)** | **RMST difference**  **(95% CI)** | **Cox models** | | |
|  |  |  | **HR (95% CI )** | **P value** | **P for interaction** |  |  |  | **HR (95% CI )** | **P value** | **P for interaction** |  |  |  | **HR (95% CI )** | **P value** | **P for interaction** |
| **Male** |  |  |  |  |  |  |  |  |  |  |  |  |  |  |  |  |  |
| Binary SLEs |  |  |  |  | 0.385 |  |  |  |  |  | 0.458 |  |  |  |  |  | 0.724 |
| No SLEs | 2.58 | 0 (reference) | 1 (reference) |  |  |  | 2.14 | 0 (reference) | 1 (reference) |  |  |  | 0.65 | 0 (reference) | 1 (reference) |  |  |
| SLEs | 2.97 | -2.51 (-3.66, -1.35) | 1.17 (1.04, 1.31) | 0.010 |  |  | 2.38 | -2.23 (-3.23, -1.23) | 1.14 (1.00, 1.30) | 0.051 |  |  | 0.88 | -0.73 (-1.35, -0.11) | 1.28 (1.02, 1.59) | 0.033 |  |
| Categorical SLEs |  |  |  |  | 0.599 |  |  |  |  |  | 0.756 |  |  |  |  |  | 0.384 |
| No SLEs | 2.58 | 0 (reference) | 1 (reference) |  |  |  | 2.14 | 0 (reference) | 1 (reference) |  |  |  | 0.65 | 0 (reference) | 1 (reference) |  |  |
| 1-2 SLEs | 2.97 | -2.45 (-3.60, -1.31) | 1.17 (1.04, 1.31) | 0.011 |  |  | 2.37 | -2.14 (-3.14, -1.15) | 1.14 (1.00, 1.29) | 0.058 |  |  | 0.89 | -0.77 (-1.39, -0.14) | 1.30 (1.04, 1.62) | 0.023 |  |
| SLEs > 2 | 2.87 | -4.01 (-8.29, 0.91) | 1.18 (0.69, 2.00) | 0.544 |  |  | 2.46 | -4.76 (-9.02, -0.50) | 1.22 (0.69, 2.17) | 0.489 |  |  | 0.41 | 0.31 (-2.36, 2.97) | 0.60 (0.15, 2.41) | 0.468 |  |
| Continuous SLEs |  |  |  |  | 0.593 |  |  |  |  |  | 0.817 |  |  |  |  |  | 0.999 |
| Per additional SLE | 2.72 | -1.62 (-2.43, -0.80) | 1.10 (1.01, 1.19) | 0.029 |  |  | 2.23 | -1.60 (-2.30, -0.89) | 1.10 (1.01, 1.21) | 0.034 |  |  | 0.73 | -0.36 (-0.80, 0.08) | 1.10 (0.94, 1.28) | 0.235 |  |
| **Female** |  |  |  |  |  |  |  |  |  |  |  |  |  |  |  |  |  |
| Binary SLEs |  |  |  |  | 0.385 |  |  |  |  |  | 0.458 |  |  |  |  |  | 0.724 |
| No SLEs | 1.99 | 0 (reference) | 1 (reference) |  |  |  | 1.60 | 0 (reference) | 1 (reference) |  |  |  | 0.56 | 0 (reference) | 1 (reference) |  |  |
| SLEs | 2.88 | -2.03 (-2.90, -1.16) | 1.22 (1.10, 1.35) | < 0.001 |  |  | 2.22 | -1.21 (-1.95, -0.46) | 1.18 (1.05, 1.32) | 0.006 |  |  | 0.90 | -0.80 (-1.28, -0.32) | 1.23 (1.02, 1.49) | 0.032 |  |
| Categorical SLEs |  |  |  |  | 0.599 |  |  |  |  |  | 0.756 |  |  |  |  |  | 0.384 |
| No SLEs | 1.99 | 0 (reference) | 1 (reference) |  |  |  | 1.60 | 0 (reference) | 1 (reference) |  |  |  | 0.56 | 0 (reference) | 1 (reference) |  |  |
| 1-2 SLEs | 2.86 | -1.93 (-2.80, -1.06) | 1.21 (1.09, 1.35) | < 0.001 |  |  | 2.20 | -1.13 (-1.87, -0.39) | 1.17 (1.04, 1.32) | 0.008 |  |  | 0.90 | -0.76 (-1.23, -0.28) | 1.22 (1.01, 1.48) | 0.040 |  |
| SLEs > 2 | 3.46 | -4.55 (-7.76, -1.34) | 1.47 (1.05, 2.06) | 0.025 |  |  | 2.69 | -3.10 (-5.84, -0.36) | 1.33 (0.91, 1.95) | 0.141 |  |  | 1.06 | -1.88 (-3.63, -0.12) | 1.46 (0.79, 2.69) | 0.223 |  |
| Continuous SLEs |  |  |  |  | 0.593 |  |  |  |  |  | 0.817 |  |  |  |  |  | 0.999 |
| Per additional SLE | 2.37 | -1.16 (-1.74, -0.58) | 1.11 (1.04, 1.19) | 0.002 |  |  | 1.87 | -0.71 (-1.21, -0.21) | 1.09 (1.02, 1.18) | 0.019 |  |  | 0.71 | -0.44 (-0.76, -0.12) | 1.11 (0.99, 1.26) | 0.086 |  |

Models were adjusted for age, sex, education, body mass index, hypertension, and diabetes.

CI, confidence interval; ELSA, English Longitudinal Study of Ageing; HR, hazard ratio; HRS, Health and Retirement Study; RMST, restricted mean survival time.


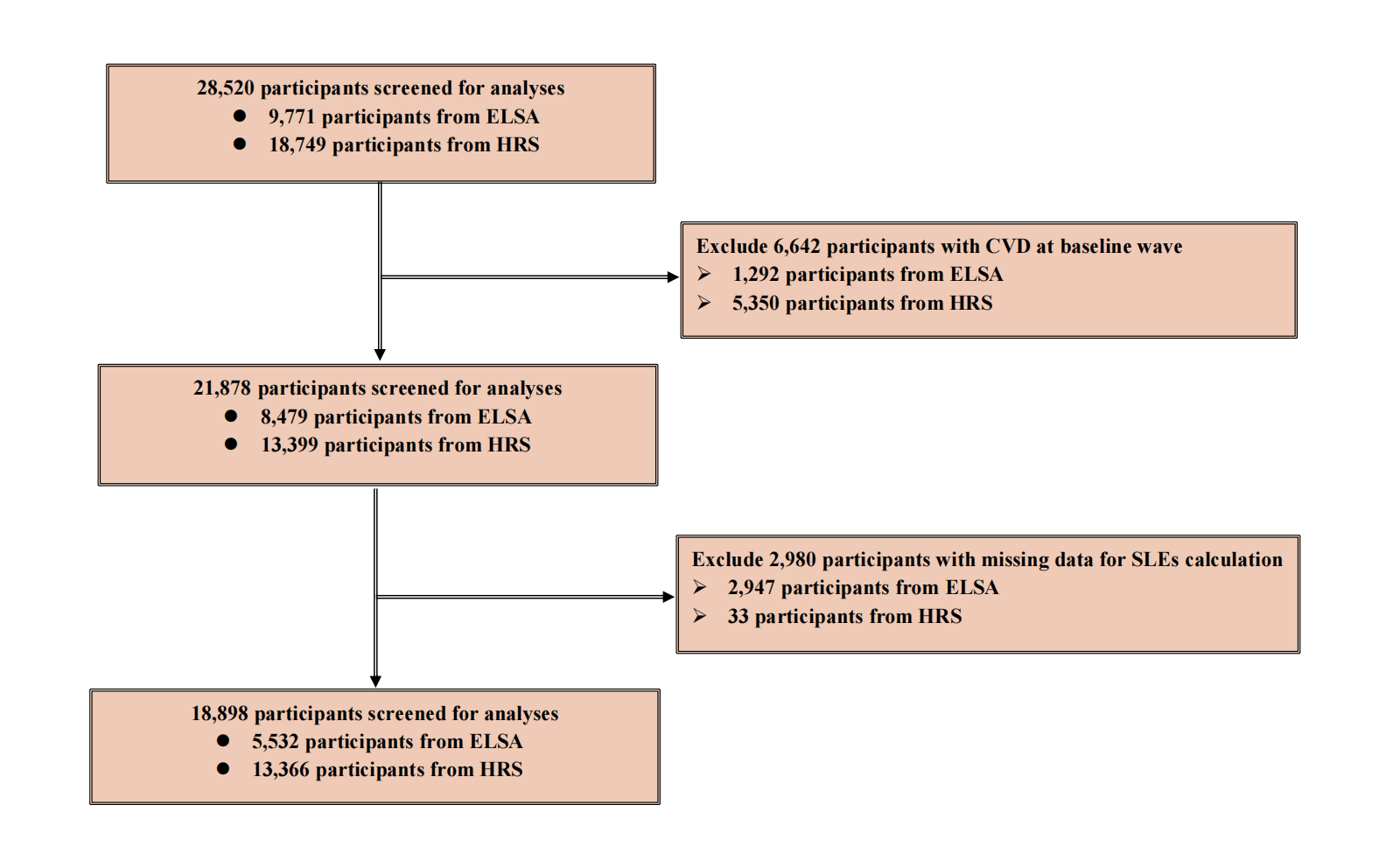


**Supplementary Figure S1. Flow chart of participant selection.**

CVD, Cardiovascular Disease; ELSA, English Longitudinal Study of Ageing; HRS, Health and Retirement Study; SLEs, stressful life events.
